# Four new genome sequences of the Pallas’s cat (*Otocolobus manul*): an insight into the patterns of within-species variability

**DOI:** 10.3389/fgene.2024.1463774

**Published:** 2024-12-09

**Authors:** Jana Bubenikova, Martin Plasil, Pamela A. Burger, Petr Horin

**Affiliations:** ^1^ Research Group Animal Immunogenomics, CEITEC – VETUNI Brno, Brno, Czechia; ^2^ Research Institute of Wildlife Ecology, University of Veterinary Medicine Vienna (VETMEDUNI), Vienna, Austria; ^3^ Department of Animal Genetics, VETUNI Brno, Brno, Czechia

**Keywords:** manul, genome, variability, EPAS1, sequencing

## Abstract

Manul (*Otocolobus manul*) is the only representative of the genus *Otocolobus,* which makes up the Leopard Cat lineage along with the genus *Prionailurus*. Their habitat is characterized by harsh environmental conditions. Although their populations are probably more stable than previously thought, it is still the case that their population size is declining. Conservation programs exist to protect manuls, but those based on captive breeding are often unsuccessful due to their increased susceptibility to diseases. The manul is therefore a suitable model species for evolutionary and diversity studies as well as for studying mechanisms of adaptation to harsh environment and mechanisms of susceptibility to diseases. Recently, the genome of the *O. manul* based on nanopore long-range sequencing has been published. Aiming to better understand inter- and intraspecific variation of the species, we obtained information on genome sequences of four other manuls, based on whole genome resequencing via the Illumina platform. On average, we detected a total of 3,636,571 polymorphic variants. Information on different types of structural variants and on the extent of SNP homozygosity, not available from the reference genome, was retrieved. The average whole-genome heterozygosity was almost identical to that found in the *O. manul* reference genome. In this context, we performed a more detailed analysis of the candidate gene *EPAS1* potentially related to adaptation to the hypoxic environment. This analysis revealed both inter- and intraspecific variation, confirmed the presence of a previously described non-synonymous substitution in exon 15 unique to manuls and identified three additional unique non-synonymous substitutions located in so far not analyzed *EPAS1* exonic sequences. The analysis of lncRNA located in the intron 7 of *EPAS1* revealed interspecific variability and monomorphic nature of the sequence among analyzed manuls. The data obtained will allow more detailed analyses of the manul genome, focusing on genes and pathways involved in their adaptation to the environment and in susceptibility to diseases. This information can be helpful for optimizing conservation programs for this understudied species.

## 1 Introduction

Manul, also called Pallas’s cat or Rock Wildcat (*Otocolobus manul*), is the only representative of the genus *Otocolobus*, which makes up the Leopard Cat lineage along with the genus *Prionailurus* (Kitchener, 2017). Their habitat range is relatively wide but fragmented. Unlike other representatives of the lineage inhabiting wetland habitats, the manul lives in inhospitable cold arid montane steppes of Central Asia. Their habitat is characterized by an extreme climate with minimal rainfall and a wide range of temperatures (Ross et al., 2020). They have been recorded at altitudes up to 5,600 m (Werhahn et al., 2018). Manuls prefer rocky areas providing shelter, completely avoiding open spaces and areas with snow cover higher than 10 cm^2^. Survival in harsh conditions is made possible by the manul’s physical characteristics. Its body structure is compact and stocky, with long shaggy fur that is double in length on its underparts - an adaptation to extremely cold winters (Ross, 2019). Short legs, low set ears and specific coat color allow the manul to blend in with its surroundings where cover options are sparse. The necessity to adapt to these conditions is probably a consequence of interspecific predation (Ross et al., 2020).

According to the IUCN Red List of endangered species, the manul is currently listed in the category “Least Concern” (Ross et al., 2020). Although their populations are probably more stable than previously thought, their current situation differs from country to country; they are classified as locally threatened in some countries and probably extinct in certain areas of its original range (Khorozyan et al., 2021). The overall population size is declining for a variety of reasons (Ross, 2019).

Individual manul populations are highly geographically fragmented, which may have negative impacts on the effective population size in terms of maintaining genomic variability. There are conservation programs in place to protect these unique cats, but information on their ecology, behavior and distribution is still rather sparse (Ross et al., 2020).

Probably due to their distinct habitat requirements, their husbandry in zoos is quite complicated and often unsuccessful despite their increasing popularity. Although diseases do not appear to be a significant threat in wild individuals, increased susceptibility to a variety of infections has been described in captive manuls (Ketz-Riley et al., 2003). Observed high mortality rates due to an acute form of toxoplasmosis appear to be a threat especially for young kittens (Brown et al., 2005; Basso et al., 2005).

The *O. manul* species is therefore valuable not only as a model for evolutionary and diversity studies, but it can also serve as a model for studying mechanisms of adaptation to harsh environment and mechanisms of resilience/susceptibility to diseases threatening other animal species and humans.

Whole genome sequencing (WGS) is an important tool for such studies not only in humans, but also in animals and plants (Prokop et al., 2018; Aylward et al., 2022; Pei et al., 2024). As it provides comprehensive base-by-base view of the genome, it is suitable for various applications not only in human genomics, but also in fields such as veterinary genomics, agrigenomics, ecology and biodiversity protection (Brlek et al., 2024; Wright et al., 2020). Recently, the genome of the *O. manul*, based on nanopore long-range sequencing of a single individual, has been published (Flack et al., 2023). To better understand inter- and intraspecific variation of the species, it is necessary to expand this information with additional resources. Using whole genome resequencing via the Illumina platform, we have obtained information on the genomes of four other manuls. It showed that all four genomes were similar in most characteristics of their variability. Information on different types of structural variants and on the extent of SNP homozygosity not available from the reference genome was retrieved. Furthermore, the four whole genome sequences, along with a recent report on unique features of the manul gene *EPAS1* (Robinson et al., 2024)*,* allowed to demonstrate how this information can be used for analyzing genes of interest, e.g., genes of adaptation.

## 2 Methods

### 2.1 Samples and DNA extraction

Tissues of four different manul individuals (one female and three males) obtained from Czech ZOOs (ZOO Jihlava, ZOO Brno, ZOO Prague) were selected based on available information about their origin and relatedness. Information on all used samples along with information on the individual used as a source of the reference genome is in Supplementary Table S1. The individuals finally selected for the sequencing are listed in Table 1. The samples were obtained either post-mortem or as part of veterinary procedures performed for other reasons and shared by respective veterinarians. Therefore, no special ethical approval was needed.

**TABLE 1:** Samples used for sequencing.

Animal Id	Place of origin	Sex	Tissue used for DNA extraction	Year of sampling
manul 4	ZOO Jihlava	male	intestine	2012
manul 5	ZOO Jihlava	male	intestine	2012
manul 7	ZOO Praha	male	intestine	2003
manul 552	ZOO Brno	female	blood	2017

DNA was extracted from available tissue samples (either blood or intestine) using Qiagen (Germany) MagAttract HMW DNA isolation kit. The kit was used according to the manufacturer’s recommendations. Two isolations were made for each individual. DNA samples were evaluated in terms of purity (absorbance) and concentration using Tecan (Switzerland) Infinite 200 Pro plate reader. DNA samples were stored at 4 C for 5 days and then transported on dry ice for sequencing. Samples were checked prior to library construction using Agilent 5,400 fragment analyzer. All samples passed QC (quantity ≥200 ng; OD260/280 = 1.8–2.0, no degradation).

### 2.2 Sequencing and bioinformatic analysis

Sequencing and subsequent whole genome bioinformatic analyses were outsourced at Novogene Europe, Cambridge, United Kingdom.

#### 2.2.1 Library construction, quality control and sequencing

A total amount of 0.2 μg DNA per sample was used as input material for the DNA library preparations. The genomic DNA sample was randomly fragmented by sonication to a size of 350 base pairs (bp). Then DNA fragments were endpolished, A-tailed, and ligated with the full-length adapter for Illumina sequencing. The fragments with adapters were size selected, PCR amplified, and purified by AMPure XP system (Beverly, United States). Subsequently, library quality was assessed on the Agilent 5,400 system (Agilent, United States) and quantified by qPCR (1.5 nM). The qualified libraries were pooled and sequenced on Illumina NovaSeq X Plus platform with PE150 chemistry.

#### 2.2.2 Bioinformatic analysis

Distribution of sequencing quality along reads and sequencing error rate were evaluated, low-quality reads and adaptors were filtered using fastp (v.0.20.0) with parameters -g -q 30 -u 50 -n 15 -L 150.

The OtoMan_p1.0 genome (GCA_028564725.2) was used as reference. The filtered sequencing data were mapped to the reference sequence through BWA (Li and Durbin, 2009) software (v0.7.17-r1188) (parameters: mem -t 4 -k 32 -M). Resulting alignments were sorted using SAMtools (v1.13) (Danecek et al., 2021) with parameters sort -@ 6 -m 2G and merged for each sample using Picard (v1.111) (Broad Institute, 2019).

Single nucleotide polymorphisms (SNP) and indels were called for entire cohort (joint calling) using Haplotypecaller from GATK (v4.0.5.1) (DePristo et al., 2011) with the following parameters--pair-hmm-gap-continuation-penalty 10 -ERC GVCF--genotyping-mode DISCOVERY -stand-call-conf 30. Polymorphisms detected were annotated using ANNOVAR (v2015Dec14) (Wang et al., 2010) and their characteristics (e.g., quality, total numbers and distribution in different genomic regions) were evaluated. The original annotation record for reference genome was kindly provided by the authors (Flack et al., 2023).

As for structural variants (SVs), BreakDancer (v1.4.4) (Chen et al., 2009) software was used with default parameters to detect indels, inversions, intra-chromosomal translocations and inter-chromosomal translocations. The SVs detected were filtered by removing those with less than 2 supporting reads; indels and inversions were further annotated by ANNOVAR. Characteristics of SVs such as their total numbers, distribution across genome and length were assessed.

Based on the genome reads depth, CNVnator (v0.3) (Abyzov et al., 2011) was used to detect Copy Number Variants (CNVs) of potential deletions and duplications with the following parameter -call 100. The CNVs detected were further annotated by ANNOVAR and their characteristics determined.

The distribution of all types of variants across the whole genomes was visualized by Circos (Krzywinski et al., 2009).

#### 2.2.3 Analysis of the *EPAS1* gene

To confirm the presence of a serine to proline substitution in exon 15, residue 806, presumably unique to manuls (Robinson et al., 2024), the *EPAS1* nucleotide sequence was examined in the four manul individuals analyzed here, in the *O. manul* reference genome and in the genomes of two related felid species, the black-footed cat and the jungle cat. They were selected for a comparison as related felid species with an available whole genome sequence (WGS), living in extreme but different habitats. The sequence XM_023251650.2 annotated in the *F. catus* reference genome (*Felis catus*_Fca126_mat1.0) was used as a BLAST (web-based, megablast mode) query to find orthologous sequences in the genomes of the manul *O. manul* (GCA_028564725.2), the black-footed cat *Felis nigripes* (GCA_0324586 15.1) and the jungle cat *Felis chaus* (GCA_019924945.1). Coordinates retrieved from the *O. manul* genome were then used to search the four newly sequenced genomes and the two other felid genomes to assess within-species variability in the gene and for the interspecific comparisons, respectively. Extracted sequences were aligned in BioEdit (7.2.5) (Hall, 1999) using the ClustalW software (Thompson et al., 1994). Inter- and intra-species nonsynonymous substitutions were identified in the inferred protein sequences and analyzed for potentially deleterious effects on protein functionality using the SIFT tool (Sim et al., 2012).

Since the *EPAS1* gene contains one predicted lncRNA (XR_006595538.1) within its seventh intron in the cat reference genome, we have employed the above-mentioned strategy to investigate this non-coding part of the *EPAS1* gene in our data as well as in *F. chaus* and *F. nigripes* genome sequences.

## 3 Results

### 3.1 Sequencing and bioinformatic analysis

#### 3.1.1 Data quality control and data filtration

The overall sequencing quality was comparable for all four samples. The total of raw data was 231.6 G bases (manul 552), 148.3 G bases (manul 7), 137.1 G bases (manul 5) and 161.8 G bases (manul 4). The percentage of clean data ranged from 90.61% to 96.29%. Percentage of Q30 bases ranged from 91.47% to 96.20%. The total number of raw read pairs were 1,543,753,528 (manul 552), 988,603,768 (manul 7), 913,880,502 (manul 5) and 1,078,790,822 (manul 4). All sequencing data statistics are available in Supplementary File S1 (sheets 01–04).

#### 3.1.2 Sequence alignment and mapping statistics

Sequencing data of individual samples aligned to the OtoMan_p1.0 reference genome with a total length of 2,487,682,665 bp showed mapping rates ranging from 99.29% to 99.72%. The average depths for samples 4, 5 and 7 ranged from 40.60X-48.00X, sample 552 quite differs in this parameter, with an average depth of 73.66X, due to higher amount of raw sequencing data. For all four samples, the values of the average depth across chromosomes as well as mapping statistics are available in Supplementary File S1 (sheet 05).

#### 3.1.3 Variant detection and annotation

##### 3.1.3.1 Single nucleotide polymorphisms

Total numbers of SNPs detected by ANNOVAR were 1,737,330; 1,736,302; 1,905,426 and 1,712,028 for samples 4, 5, 7 and 552, respectively. Numbers of SNPs based on their localization within genome and genome-wide heterozygous rates are in Table 2. The frequency of each SNP mutation type (e.g., T:A>C:G) is available in Supplementary File S2 (sheet 01).

**TABLE 2:** Numbers of SNPs detected using ANNOVAR.

SNP type		Sample			
		4	5	7	552
Upstream		17,361	16,964	19,839	16,738
Exonic	Stop gain	121	125	196	115
	Stop loss	13	15	22	11
	Synonymous	4,516	4,411	5,337	3,997
	Non-synonymous	5,860	5,662	8,460	5,244
Intronic		304,742	302,111	336,810	301,549
Splicing		213	217	273	213
Downstream		16,530	16,607	18,559	16,040
Intergenic		1,378,329	1,380,654	1,501,951	1,358,891
Transitions		1,137,479	1,137,863	1,198,751	1,123,572
Transversions		599,851	598,439	706,675	588,456
Transitions/transversions ratio		1.896	1.901	1.696	1.909
Heterozygous rate (%)		0.0442	0.0445	0.0504	0.0444
Total		1,737,330	1,736,302	1,905,426	1,712,028

Characteristics of SNP quality distribution (distribution of SNP support reads number, distribution of distances between adjacent SNPs and the cumulative distribution of SNP quality) are available in Supplementary File S2 (sheet 01). The results are similar among samples except for the cumulative distribution parameter, where the SNPs of sample 7 are of higher quality. SNP densities along chromosomes for all 4 samples are also available in Supplementary File S2 (sheet 01).

##### 3.1.3.2 Indels

Total numbers of indels detected by GATK were 1,777,420; 1,782,381; 1,915,888 and 1,739,717 for samples 4, 5, 7 and 552, respectively. Numbers of indels according to their localization within genome and genome-wide heterozygous rates are in Table 3. The results are similar for all samples.

**TABLE 3:** Numbers of indels detected using GATK.

Indel type		Sample			
		4	5	7	552
Upstream		16,442	16,399	18,418	16,122
Exonic	Stop gain	33	31	69	33
	Stop loss	7	8	12	7
	Frameshift deletion	1,151	1,133	1,417	1,098
	Frameshift insertion	987	964	2,641	932
	Non-frameshift deletion	301	307	342	282
	Non-frameshift insertion	174	171	1,176	157
Intronic		327,913	328,438	354,884	320,743
Splicing		184	190	294	185
Downstream		16,586	16,537	18,164	16,132
Upstream/Downstream		1,411,274	1,415,823	1,512,555	1,381,735
Intergenic		528,740	528,719	684,734	518,787
Insertion		1,233,699	1,239,039	1,217,005	1,203,978
Deletion		327,913	328,438	354,884	320,743
Heterozygous rate (%)		0.0417	0.0432	0.0439	0.0353
Total		1,777,420	1,782,381	1,915,888	1,739,717

Length distributions of indels located within coding sequences and indel densities along chromosomes for all 4 samples are available in Supplementary File S2 (sheet 02).

##### 3.1.3.3 Structural variants

Mutations of the size >50 bp were considered as structural variants (SVs) (Huddleston et al., 2017). Total numbers of SVs detected by BreakDancer and annotated by ANNOVAR were 50,340; 39,846; 57,157 and 41,860 for sample 4, 5, 7 and 552, respectively. Numbers of SVs categorized according to their type and localization within genome are in Table 4.

**TABLE 4:** Numbers of SVs detected using BreakDancer and ANNOVAR.

SV type	Sample			
	4	5	7	552
Upstream	226	204	245	225
Exonic	955	938	1,065	655
Downstream	192	192	157	182
Intronic	5,297	4,760	5,284	5,202
Upstream/Downstream	1	2	3	2
Intergenic	23,953	21,869	23,613	24,380
Splicing	6	14	8	14
Insertion	8	14	3	206
Deletion	30,035	27,437	29,684	30,016
Inversion	587	528	688	438
Intra-chromosomal translocations	936	799	1,397	1,884
Inter-chromosomal translocations	18,774	11,068	25,385	9,316
Total	50,340	39,846	57,157	41,860

**FIGURE 1:**
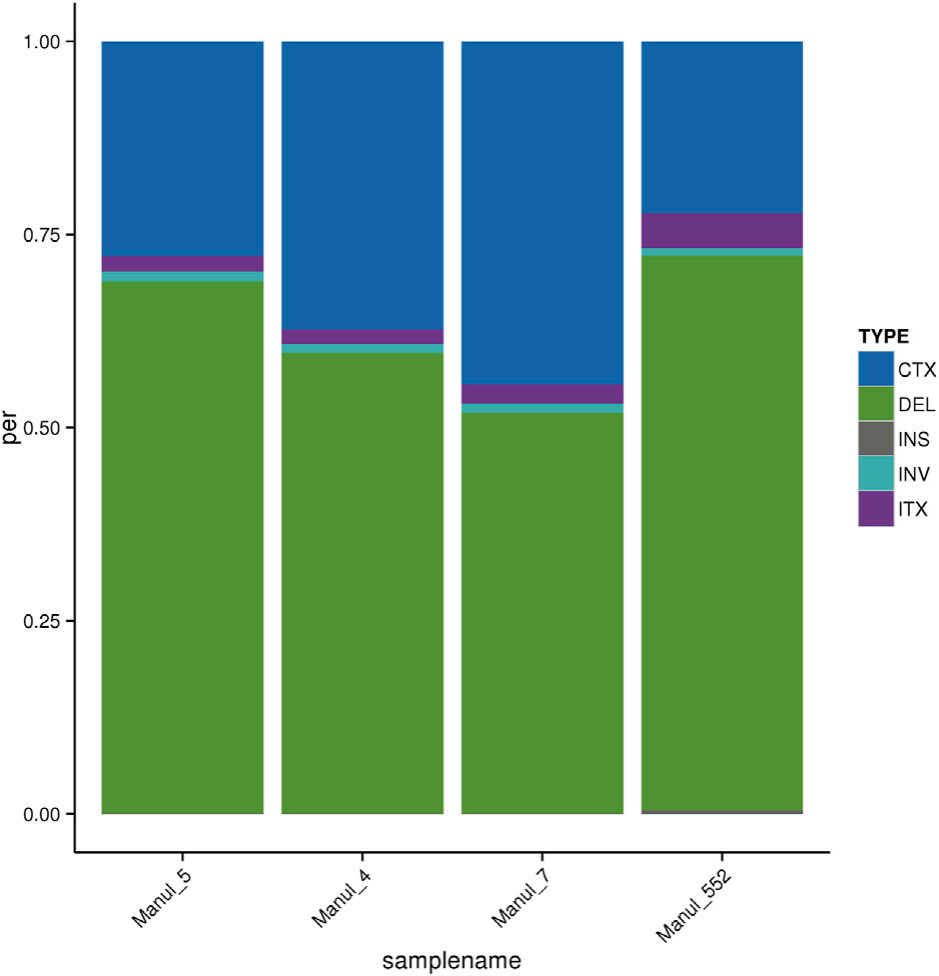
Distribution of SVs according to their types. CTX–Inter-chromosomal translocation, DEL–Deletion, INS–Insertion, INV–Inversion, ITX–Intra-chromosomal translocation.

For SVs, the results are similar for most parameters, main variation being a relative proportion of deletions and inter-chromosomal translocations (Figure 1). The length distribution of SVs is available in Supplementary File S2 (sheet 03).

##### 3.1.3.4 Copy number variants

Total numbers of CNVs detected by CNVnator and annotated by ANNOVAR were 14,615; 11,139; 8,933 and 15,900 for sample 4, 5, 7 and 552, respectively. Numbers of CNVs categorized according to their type and localization in genomic regions and total duplication/deletion lengths are in Table 5.

**TABLE 5:** Numbers of CNVs detected using CNVnator and ANNOVAR.

CNV type	Sample			
	4	5	7	552
Upstream	173	130	96	182
Exonic	765	771	1,448	998
Intronic	1,939	1,344	667	2,222
Downstream	106	81	60	123
Upstream/Downstream	3	1	0	6
Intergenic	11,626	8,811	6,662	12,368
Duplication number	1,922	1,684	4,372	914
Deletion number	12,693	9,455	4,561	14,986
Duplication length (bp)	18,150,600	17,789,100	48,158,900	7,303,100
Deletion length (bp)	15,569,000	14,824,500	15,253,700	14,478,200
Total	14,615	11,139	8,933	15,900

**FIGURE 2:**
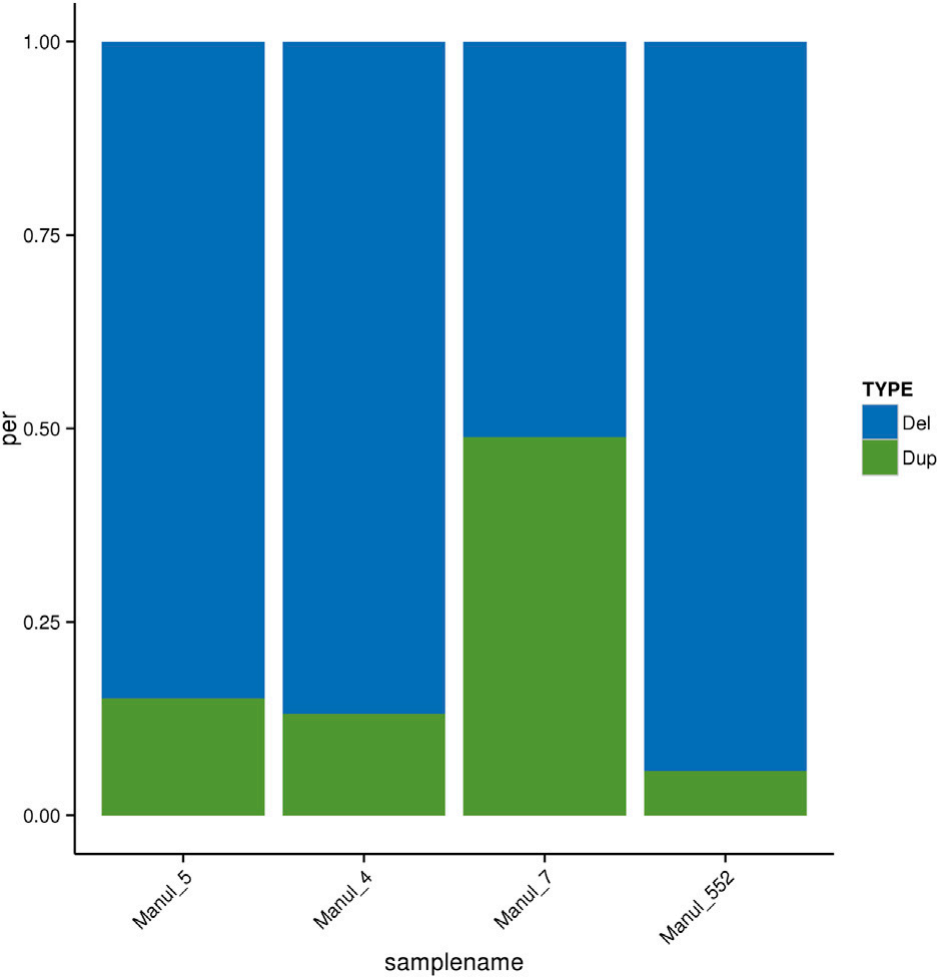
Distribution of CNV types. Del–CNVs with decreased copy number, Dup–CNVs with increased copy number.

The total numbers of CNVs are lower for the sample 7, which also has a different distribution within CNV types, with a higher number of duplications at the expense of the number of deletions. This deviation can be seen in Figure 2.

##### 3.1.3.5 Whole genome variations distribution

All the variant types described above are visualized together at the genome-wide level in Supplementary Figures S1A-D.

##### 3.1.3.6 Genome-wide heterozygosity

The average genome-wide heterozygosity was calculated to be 0.046% when only SNPs were considered, 0.087% when SNPs were combined with indels. Comparison with the reference genome and values of the four samples individually are given in Table 6.

**TABLE 6:** Genome-wide heterozygosity of the OtoMan_p1.0 reference genome and our four samples.

	Heterozygous SNPs	Heterozygous SNPs + indels	Genome-wide heterozygosity (SNPs)	Genome-wide heterozygosity (SNPs + indels)	Average number of heterozygous SNPs per 1 Mb
OtoMan_p1.0	1,184,174	2,045,611	0.00048	0.00082	476
Manul 4	1,099,556	2,136,919	0.00044	0.00086	442
Manul 5	1,107,019	2,181,698	0.00045	0.00088	445
Manul 7	1,253,792	2,345,885	0.00050	0.00094	504
Manul 552	1,104,531	1,982,683	0.00044	0.00080	444
Manuls 4, 5, 7 and 552 on average	1,141,224	2,161,796	0.00046	0.00087	459

#### 3.1.4 Analysis of the *EPAS1* gene

##### 3.1.4.1 Interspecific comparisons

The total length of the *EPAS1* coding sequence of the domestic cat is 2,619 bp. Compared to the reference genome of the domestic cat, the first 24 bp sequence in exon one was not found in any of the analyzed manul and the *F. nigripes* nor in *F. chaus* genomes.

The non-synonymous substitution CCC→TCC in exon 15, residue 808 as compared to the *F. catus* protein sequence (numbered as residue 806 in the original publication where compared to the human protein sequence (Robinson et al., 2024)) was found in all five manul genomes analyzed; it was found neither in *F. nigripes* nor in *F. chaus*.

Total numbers of nucleotide positions differing between all possible combinations of the species analyzed are shown in Table 7. All alignments are available in Supplementary File S3.

**TABLE 7:** Numbers of nucleotide (green) and amino acid (blue) positions differing between species in the *EPAS1* coding and protein sequences.

	*Felis catus*	*Otocolobus manul*	*Felis chaus*	*Felis nigripes*
*Felis catus*		20^a^	12^a^	15^a^
*Otocolobus manul*	7^a^		24	27
*Felis chaus*	4^a^	5		19
*Felis nigripes*	6^a^	7	4	

^a^ The deletion of 24 bp/8 amino acids considered as one difference.

A comparison of *in silico* translated EPAS1 protein sequences from the four felid species analyzed (including the domestic cat) showed four non-synonymous substitutions found in manuls only (N84H, S446G, G611S and the P808S). Two additional non-synonymous substitutions were present in the wild felids compared to the domestic cat (G372S and T659A). Three non-synonymous substitutions were unique to the black-footed cat (V654A, V703L and M776I) and one for the jungle cat (A574V). As it is clear from nucleotide sequences, all manuls, the black-footed and jungle cat lacked the first eight amino acids of EPAS1 compared to the domestic cat. Total numbers of amino acid positions differing between all possible combinations of the species analyzed are shown in Table 7.

Most of the non-synonymous substitutions detected were scored as tolerated by SIFT with two exceptions; substitutions N84H in EPAS1 unique to manuls and A574V unique to the jungle cat were scored as affecting the protein function.

The analysis of lncRNA present in the *EPAS1* intron 7 revealed a total of 27 SNPs, 6 indels and one SV (a 233 bp sequence found only in the domestic cat). Out of these, 9 SNPs and 3 indels were unique for manuls. The alignment is available in Supplementary File S3.

##### 3.1.4.2 Within-species EPAS1 variability in manuls

The coding sequence of *EPAS1* showed no SNPs. *EPAS1* 5′ UTR exhibited single polymorphic deletion of T at position 222 and the 3′ UTR showed single polymorphic insertion at position 1,405 (G). All newly sequenced samples shared both the insertion and the deletion and the polymorphism was observed between our cohort and reference genome. All alignments are available in Supplementary File S3.

The lncRNA was monomorphic in all our manul sequences.

Alignment files for both *EPAS1* sequences and lncRNA are available in the Supplementary File S4.

## 4 Discussion

Genomes of organisms adapted to specific environments may provide important information on their evolution and diversity, mechanisms of adaptation and/or resilience to disease. Specific adaptations to various environments were reported for various species, such as adaptation to high altitude in deer mice (Storz and Scott, 2021; Schweizer et al., 2019) or ruminants (Friedrich and Wiener, 2020), adaptation to hypoxic and cold underground environment in mole rat (Faulkes et al., 2024), longevity and unique immune adaptations in bats (Jebb et al., 2020), trypanosome tolerance (Naessens et al., 2002) or heat stress and water-deprivation stress tolerance in livestock living in tropic or arid areas (Kaushik et al., 2023; Cao et al., 2019). Information retrieved from whole genome sequences may then be used in conservation programs for endangered species as well as for optimizing environments of captive individuals (Wright et al., 2020; van der Valk et al., 2024; Brandies et al., 2019; Byrne et al., 2021). Considering the extreme climate resulting in a harsh cold and arid habitat of manuls in Central Asia, their genomes and their genetic variability is assumed to reflect the manuls’ adaptation to these specific conditions. The importance of such adaptation is illustrated by demands of keeping them in captivity (Ketz-Riley et al., 2003; Brown et al., 2005).

So far, only one whole genome sequence of *O. manul* has been available (OtoMan_p1.0, GCA_028564725.2). Four individual whole genome sequences obtained in this study were aligned based on this reference genome sequenced by the long-read Nanopore technology. They provide additional information on intraspecific variability, which is not available from the current reference genome assembly. As the parameters characterizing the quality of sequencing were within the standard range, the data obtained may be considered as reliable. However, the short-read sequencing technique used here has some limitations. Due to their short length, the reads may not be always successfully mapped to specific regions of the reference genome, some of them may be discarded, which may result in gaps in the sequences. The accuracy of the detection of pseudogenes, sequencing of repetitive regions, and of CNV identification is generally lower. Short-read sequences are of limited use especially in the analysis of complex genomic regions such as MHC or NKR regions, which need manual annotation (Peel et al., 2022). Despite these disadvantages, individual genomes obtained by short-read sequencing are still thoroughly explored as a useful source of additional information, especially when a long-read based reference genome is available; for example, to determine the level of diversity and signatures of selection in endangered species, which may be of use for conservation programs (Wright et al., 2020; Xia et al., 2021) or to search for loci associated with adaptation (Peng et al., 2023; Lee et al., 2018; Yu et al., 2016) or disease susceptibility (Meurs et al., 2021).

All four genomes analyzed here were similar amongst them in most characteristics. This is probably due to a sampling bias caused by limited availability of this type of samples. Tissues used for DNA extraction (manuls 4, 5 and 7) were necropsy samples originally collected for diagnostic purposes. The blood sample (manul 552) was collected for diagnostic purposes as well. The samples originated from three Czech zoos; within a zoo, the animals were related. Therefore, out of seven samples originally available, four different manul individuals (one female and three males) were selected based on available information about their origin and relatedness, with manuls 4 and 5 being littermates. Despite this primary limitation of sampling, the data obtained extend our knowledge on the overall genomic variability as well as of the structure of the individual variation represented by different types of genetic polymorphism. This information cannot be retrieved from the single reference genome OtoMan_p1.0.

We anticipated to obtain more information on the overall extent of genetic polymorphism of the species. In fact, in our four individuals sequenced, we detected a total of 3,636,571 polymorphic variants of all types on average (the mean numbers were 1,772,772 for SNPs, 1,803,852 for indels, 47,301 for SVs, and 12,647 for CNVs per individual). For comparison, 2,694,148 variants were identified in the OtoMan_p1.0 reference genome, most variants being single nucleotide variants (SNVs) and small insertions and deletions (Flack et al., 2023).

The difference in the proportion of deletions and intra-chromosomal translocations observed in manul 552 can be explained partly by its different origin, but also to a higher depth of sequencing in that individual (average depth 73.66X compared to an average of 43.38X in the others), leading to an improved detection of credibly identified variants (Sims et al., 2014). Slighter differences in the number of indels can be attributed to the varying degree of relatedness between individuals; sibling manuls 4 and 5 are more similar to each other compared to the other two.

Average whole-genome heterozygosity is another parameter, for which information from more than one reference genome allow its more accurate estimation. The average whole-genome heterozygosities of both SNP/indels and SNPs alone were almost identical between our samples and the *O. manul* reference genome. The average number of heterozygous SNPs per 1 Mb was slightly higher in the reference genome compared to the average of the newly sequenced manuls. Altogether, this limited dataset indicated moderate diversity of the genomes analyzed. Our estimate of whole-genome heterozygosity is limited not only by small sample sizes, but potentially also by other biological and non-biological factors, such as population structure or settings of bioinformatic analyses (rare allele filtering), respectively, that must be taken into account (Schmidt et al., 2021). Nevertheless, it does not contradict data on genetic diversity determined by genotyping 29 microsatellite loci on six wild manuls and ten manuls from a zoo-managed population, the former group showing slightly more genetic diversity (Robinson et al., 2024). The average SNP heterozygosity was 0.64 in our newly sequenced samples and the average heterozygosity of 29 microsatellite markers in wild and captive manuls was 0.54 and 0.59, respectively. Our small and partly related zoo-managed group is supposed to represent only a limited extent of existing variability both in captive and wild animals. On the other hand, taken together with the microsatellite data suffering from similar limitations (Robinson et al., 2024), the so far available data suggest that estimated nuclear heterozygosity levels of manuls as species are somewhere in the middle of the scale of mammalian species compared by Westbury et al. (Westbury et al., 2018), close to the panda.

However, at this stage of our knowledge, no conclusion on the diversity and genome-based conservation status of the manul captive and wild populations can be made, which emphasizes the need for extending genomic analyses of this understudied species. In a broader context, it should be kept in mind that the degree of vulnerability does not always correspond to the average heterozygosity of the genome (Westbury et al., 2018). Information on genome variability is thus important, but not sufficient on its own to decide on the IUCN red-list status and further management of a species (Schmidt et al., 2023). With the in-depth analysis of the four new manul genomes made available here, we add to the genomic resources of the species, which will be useful for future metapopulation studies about genome-wide diversity, genetic load and (captive) population management. Based on the IUCN Cat Specialist Group special issue on Pallas’s cat conservation (Barclay et al., 2019), manuls have not yet been integrated into any formal national action plan within their range countries, although strategic plans on the sustainable management exist. As such, more genomic information directly adds to the greater international visibility and credibility of conservation efforts in manuls as demanded by the authors. This will increase the potential to implement manul management actions into the National Biological Strategies and Action Plans that have been developed by 14 of the 16 range countries as an obligation to the Conservation on Biology Diversity (CBD 2019).

The nonsynonymous serine to proline substitution in exon 15, residue 806, within the hypoxia-related *EPAS1* (endothelial PAS domain protein 1) gene was found in manuls, but not in the genomes of domestic cats or snow leopards. This is a first example of a candidate gene that could be studied in the context of manuls’ adaptation, in this particular case to hypoxic environments (Robinson et al., 2024). Our data confirmed the presence of the CCC→TCC substitution in all manuls analyzed, and its absence from the genomes of the jungle cat and black-footed cat. A BLAST search of other felid NCBI genomes did not reveal it in any other felid genome suggesting that it is really unique for this species. The study by Robinson et al. (2024) focused on variation of two exons. Our analysis covered the complete CDS and identified three additional non-synonymous substitutions unique for manuls: N84H in exon 3, S446G in exon 10 and G611S in exon 13. When we compared whole *EPAS1* genomic sequences retrieved from genomes of the two felid species living in extreme but different habitats (Gray, 2014; Sliwa et al., 2013), the first part (24 bp) of the sequence annotated in the domestic cat reference genome was missing in manuls as well as in *F. chaus* and *F. nigripes*. This was then confirmed, and further variation of this part of the *EPAS1* genomic sequence was observed in some of the available annotated felid sequences at NCBI. Most available felid species carry a complete *EPAS1* sequence similar to the one annotated in the *F. catus* reference genome. Besides the three species analyzed here, variation in the length of first part of the sequence was also observed in *Panthera uncia* and *Puma concolor*. Considering the quality of annotation of most of the genomes analyzed, it is probably not an error in the annotations. Perhaps alternative splicing could be an explanation of these findings (Verta and Jacobs, 2022).

We found no polymorphic sites within the manul *EPAS1* coding sequences, suggesting possible effects of purifying selection (Nielsen, 2005). Although according to SIFT, the species-specific CCC→TCC substitution should not affect the EPAS1 protein function, another non-synonymous substitution leading to interspecific differences and potentially affecting function (N84H) was identified in the manul *EPAS1* gene, but not found in the two other species analyzed. Nevertheless, the importance of the interspecific variation and of the 5′UTR and 3′UTR within species variation of the *EPAS1* gene remain unknown. Mutations in *EPAS1* are also known in other species adapted to hypoxic environments (Lee, 2024). In human populations living in high altitudes, a reduced expression of hypoxia-inducible factor (HIF) genes protecting the body from negative effects of prolonged hypoxia, more precisely from negative effects of the mechanisms activated during hypoxia, was observed (Bigham and Lee, 2014). Interestingly, different mutations resulting in the same phenotype were observed among human populations. A mutation in the protein coding sequence was observed in the Andean population, whereas a non-coding regulatory element SNPs were identified in the Tibetan population (Lawrence et al., 2024). These findings suggest that in manuls, the non-synonymous substitutions could play a similar role in the adaptation to the high-altitude environment. In this study, we observed inter-specific and within-species variation in 5′, 3′ as well as in intronic sequences of *EPAS1*. The unique variants found in manuls’ lncRNA could have a significant regulatory function, similar to the situation found in the Tibetan population. At this stage however, we may only speculate about their functional importance. Expression studies are necessary to address these issues.

## 5 Conclusion

Altogether, this study brings the first insight into the within-species genomic variability of manuls and shows that individual whole genome sequences may serve as novel resources for studying mechanisms of adaptation and other special phenotypes characteristic for the species, which was demonstrated on an example of *EPAS1* gene and lncRNA. Although the primary aim of this study was to identify genomic sequences contributing to within-species variation, we reflected first data on individual variation of both wild and captive manuls, including a newly identified non-synonymous substitution in the *EPAS1* gene (Chen et al., 2009) to illustrate the potential of exploring genomes of specifically adapted species and of their comparisons with other species. The study thus underlines the importance of generating further genomic data on this understudied but in many aspects interesting species.

## Data Availability

The datasets presented in this study can be found in online repositories. The names of the repository/repositories and accession number(s) can be found below: https://www.ncbi.nlm.nih.gov/bioproject/, PRJNA1098449 https://www.ncbi.nlm.nih.gov/sra/, SRR28606170, SRR28606171, SRR28606172, SRR28606173, SRR28606174, SRR28606175, SRR28606176, SRR28606177 https://datadryad.org/stash, DOI: 10.5061/dryad.pzgmsbcvt.
