# Supplementary material for: Four new genome sequences of the Pallas’s cat (Otocolobus manul): an insight into the patterns of within-species variability
Source: Front Genet. 2024 Dec 9;15:1463774. doi: 10.3389/fgene.2024.1463774 (PMC11667119; doi:10.3389/fgene.2024.1463774)
Supplement: Supplementary file 1 [file DataSheet1.zip › Supplementary Material/Captions.docx]

**Supplementary Table 1:** Sample information

**Supplementary File 1:** Sequencing

**Supplementary File 2:** Variants

**Supplementary File 3:** *EPAS1* CDS (A), protein (B), UTR (C, D) and intron 7 lncRNA (E) alignments. Identical manul sequences are collapsed. In case of intraspecific variability O manul alt sequence is used to show variable sites.

**Supplementary File 4:** Alignments from SF3 presented in fasta format.

**Supplementary Figure 1**: Whole genome variations distribution in manul 4 (A), 5 (B), 7 (C) and 552 (D); the circles from outer to inner represent chromosome, SNPs, indels, CNV duplications, CNV deletions, SV insertions, SV deletions, SV inversions, SV intra-chromosomal translocations and SV inter-chromosomal translocations.
