# Supplementary material for: Four new genome sequences of the Pallas’s cat (Otocolobus manul): an insight into the patterns of within-species variability
Source: Front Genet. 2024 Dec 9;15:1463774. doi: 10.3389/fgene.2024.1463774 (PMC11667119; doi:10.3389/fgene.2024.1463774)
Supplement: Supplementary file 1 [file DataSheet1.zip › Supplementary Material/Supplementary File 3.pdf]

A) *EPAS1* CDS

```
                                    10        20        30        40        50        60        70        80        90       100
                            ....|....|....|....|....|....|....|....|....|....|....|....|....|....|....|....|....|....|....|....|
EPAS1 CDS Felis catus       ATGACAACTGACAAGGAAAAGAAAAGGAGCAGCTCGGAGAGGAGGAAGGAGAAGTCCCGGGATGCTGCCCGGTGCCGACGGAGCAAGGAGACGGAGGTCT
EPAS1 CDS Otocolobus manul  ------------------------............................................................................
EPAS1 CDS Felis chaus       ------------------------.......................A....................................................
EPAS1 CDS Felis nigripes    ------------------------............................................................................

                                   110       120       130       140       150       160       170       180       190       200
                            ....|....|....|....|....|....|....|....|....|....|....|....|....|....|....|....|....|....|....|....|
EPAS1 CDS Felis catus       TCTACGAGCTGGCCCACGAGCTGCCCCTGCCTCACAGCGTGAGCTCCCACCTGGACAAAGCCTCCATCATGCGGCTGGCCATCAGCTTCCTGCGCACACA
EPAS1 CDS Otocolobus manul  ...................A................................................................................
EPAS1 CDS Felis chaus       ....................................................................................................
EPAS1 CDS Felis nigripes    ....................................................................................................

                                   210       220       230       240       250       260       270       280       290       300
                            ....|....|....|....|....|....|....|....|....|....|....|....|....|....|....|....|....|....|....|....|
EPAS1 CDS Felis catus       CAAGCTCCTGTCCTCAGTTTGCTCTGAAAATGAGTCTGAAGCTGAGGCTAACCAACAGATGGACAACTTGTACCTGAAAGCGTTGGAGGGCTTCATTGCC
EPAS1 CDS Otocolobus manul  .................................................C........................................T.........
EPAS1 CDS Felis chaus       ....................................................................................................
EPAS1 CDS Felis nigripes    ....................................................................................................

                                   310       320       330       340       350       360       370       380       390       400
                            ....|....|....|....|....|....|....|....|....|....|....|....|....|....|....|....|....|....|....|....|
EPAS1 CDS Felis catus       GTGGTGACCCAAGATGGCGACATGATCTTTCTATCGGAAAACATCAGCAAGTTCATGGGACTCACACAGGTGGAGCTAACAGGACACAGTATCTTTGACT
EPAS1 CDS Otocolobus manul  .............................C..C...................................................................
EPAS1 CDS Felis chaus       ....................................................................................................
EPAS1 CDS Felis nigripes    ....................................................................................................

                                   410       420       430       440       450       460       470       480       490       500
                            ....|....|....|....|....|....|....|....|....|....|....|....|....|....|....|....|....|....|....|....|
EPAS1 CDS Felis catus       TCACTCATCCCTGTGACCACGAGGAGATTCGTGAGAACCTGAGTCTCAAAAATGGCTCTGGTTTTGGGAAGAAAGGCAAAGACATGTCCACAGAGCGGGA
EPAS1 CDS Otocolobus manul  ....................................................................................................
EPAS1 CDS Felis chaus       ....................................................................................................
EPAS1 CDS Felis nigripes    ....................................................................................................

                                   510       520       530       540       550       560       570       580       590       600
                            ....|....|....|....|....|....|....|....|....|....|....|....|....|....|....|....|....|....|....|....|
EPAS1 CDS Felis catus       CTTCTTCATGAGGATGAAGTGCACCGTCACCAACAGAGGCCGGACCGTCAACCTCAAGTCAGCCACCTGGAAGGTCTTGCACTGCACCGGCCAGGTGAAG
EPAS1 CDS Otocolobus manul  ....................................................................................................
EPAS1 CDS Felis chaus       ....................................................................................................
EPAS1 CDS Felis nigripes    .............................................T......................................................

                                   610       620       630       640       650       660       670       680       690       700
                            ....|....|....|....|....|....|....|....|....|....|....|....|....|....|....|....|....|....|....|....|
EPAS1 CDS Felis catus       GTCTACAACAGCTGCCCCCCTCACAGTAGTCTGTGCAGCTTCAAGGAGCCCCTGCTCTCCTGCCTCATCATCATGTGCGAACCCATCCAGCACCCATCCC
EPAS1 CDS Otocolobus manul  ....................................................................................................
EPAS1 CDS Felis chaus       ....................................................................................................
EPAS1 CDS Felis nigripes    ....................................................................................................

                                   710       720       730       740       750       760       770       780       790       800
                            ....|....|....|....|....|....|....|....|....|....|....|....|....|....|....|....|....|....|....|....|
EPAS1 CDS Felis catus       ACATGGACATCCCCCTGGACAGCAAGACCTTCCTGAGCCGCCACAGCATGGACATGAAGTTCACCTACTGTGACGACAGAATCACAGAACTGATTGGTTA
EPAS1 CDS Otocolobus manul  .........................................................................T..........................
EPAS1 CDS Felis chaus       ....................................................................................................
EPAS1 CDS Felis nigripes    ....................................................................................................

                                   810       820       830       840       850       860       870       880       890       900
                            ....|....|....|....|....|....|....|....|....|....|....|....|....|....|....|....|....|....|....|....|
EPAS1 CDS Felis catus       CCACCCTGAGGAGCTGCTCGGCCGCTCAGCCTACGAGTTCTACCATGCCCTGGACTCAGAGAACATGACCAAAAGTCACCAGAACCTGTGCACCAAAGGT
EPAS1 CDS Otocolobus manul  .................................T..................................................................
EPAS1 CDS Felis chaus       ....................................................................................................
EPAS1 CDS Felis nigripes    ....................................................................................................

                                   910       920       930       940       950       960       970       980       990      1000
                            ....|....|....|....|....|....|....|....|....|....|....|....|....|....|....|....|....|....|....|....|
EPAS1 CDS Felis catus       CAGGTGGTGAGCGGCCAGTACCGGATGCTTGCAAAGCATGGGGGCTACGTGTGGCTGGAGACCCAGGGGACGGTCATCTACAACCCTCGCAACCTGCAGC
EPAS1 CDS Otocolobus manul  ....................................................................................................
EPAS1 CDS Felis chaus       ....................................................................................................
EPAS1 CDS Felis nigripes    ....................................................................................................

                                  1010      1020      1030      1040      1050      1060      1070      1080      1090      1100
                            ....|....|....|....|....|....|....|....|....|....|....|....|....|....|....|....|....|....|....|....|
EPAS1 CDS Felis catus       CCCAGTGCATCATGTGTGTGAACTATGTCCTGAGCGAGATTGAGAAGAACGACGTGGTGTTCTCCATGGACCAGACGGAATCCCTGTTCAAGCCGCACCT
EPAS1 CDS Otocolobus manul  ............................................................................A.......................
EPAS1 CDS Felis chaus       ..........................................................T.........................................
EPAS1 CDS Felis nigripes    ..............................................................................................A.....

                                  1110      1120      1130      1140      1150      1160      1170      1180      1190      1200
                            ....|....|....|....|....|....|....|....|....|....|....|....|....|....|....|....|....|....|....|....|
EPAS1 CDS Felis catus       GACGGCCATGAGCGGCATCTTTGACAGCAGCAGTGAGGTGGCTGTGTCTGAGCAGAGTGACTACCTGTTCACCAAGCTGAAGGAGGAGCCGGAGGAGCTG
EPAS1 CDS Otocolobus manul  .............A......................................................................................
EPAS1 CDS Felis chaus       .............A......................................................................................
EPAS1 CDS Felis nigripes    .............A......................................................................................

                                  1210      1220      1230      1240      1250      1260      1270      1280      1290      1300
                            ....|....|....|....|....|....|....|....|....|....|....|....|....|....|....|....|....|....|....|....|
EPAS1 CDS Felis catus       GCCCAGCTGGCCCCCACTGCGGGGGATGCCATCATTGCCCTGGATTTCGGAAGCCAGAACTTTGAGAAGTCCTCAGCCTACAGCAGTGCCCTTCTGCCCC
EPAS1 CDS Otocolobus manul  ..................................................G.................................................
EPAS1 CDS Felis chaus       ..............T.....................................................................................
EPAS1 CDS Felis nigripes    ....................A...............................................................................

                                  1310      1320      1330      1340      1350      1360      1370      1380      1390      1400
                            ....|....|....|....|....|....|....|....|....|....|....|....|....|....|....|....|....|....|....|....|
EPAS1 CDS Felis catus       CGAGCCAGCCGTGGTCCAGAGAGCTGAGGAGCCACAGCACCCAGAGTGAGGCCGGGAGCCTGCCCGCCTTCACCGTGCCCCAAGCGGCTGCCTCCGGGAG
EPAS1 CDS Otocolobus manul  ...................................G..........C.....................................................
EPAS1 CDS Felis chaus       ....................................................................................................
EPAS1 CDS Felis nigripes    ....................................................................................................
```

```
                                     1410      1420      1430      1440      1450      1460      1470      1480      1490      1500
                            ....|....|....|....|....|....|....|....|....|....|....|....|....|....|....|....|....|....|....|....|
EPAS1 CDS Felis catus       CGCCACCCCAAGTGCCACCAGCAGCAGCAGCTGCTCCACGCCCAGCAGCCCCGGAGACTATTACACATCTCTGAATGACGATCTGAAGATTGAAGTGATC
EPAS1 CDS Otocolobus manul  ...................................................T................................................
EPAS1 CDS Felis chaus       ....................................................................................................
EPAS1 CDS Felis nigripes    ...............................................................T....................................

                                     1510      1520      1530      1540      1550      1560      1570      1580      1590      1600
                            ....|....|....|....|....|....|....|....|....|....|....|....|....|....|....|....|....|....|....|....|
EPAS1 CDS Felis catus       GAGAAGCTCTTCACCATAGATACAGAGGCAAAGGACCAGGGCAGCACCCAGACGGACTTCAGTGAGCTGGACTTGGAGACCCTGGCCCCCTACATCCCCA
EPAS1 CDS Otocolobus manul  ....................................................................................................
EPAS1 CDS Felis chaus       ....................................................................................................
EPAS1 CDS Felis nigripes    ....................................................................................................

                                     1610      1620      1630      1640      1650      1660      1670      1680      1690      1700
                            ....|....|....|....|....|....|....|....|....|....|....|....|....|....|....|....|....|....|....|....|
EPAS1 CDS Felis catus       TGGACGGGGAAGACTTCCAGCTCAGTCCCATCTGCCCCGAGGAGAGGCTTCTGCAGGAGAAGCCCCAGTCGACCCCCCAGCACTGCTTCAGCACCATGAC
EPAS1 CDS Otocolobus manul  ....................................................................................................
EPAS1 CDS Felis chaus       ....................................................................................................
EPAS1 CDS Felis nigripes    ........................................................................................T...........

                                     1710      1720      1730      1740      1750      1760      1770      1780      1790      1800
                            ....|....|....|....|....|....|....|....|....|....|....|....|....|....|....|....|....|....|....|....|
EPAS1 CDS Felis catus       GAACATCTTCCAGCCGCTGGCGCCCGGGGCCTCCCACAGCCCTTTCCTCCTGGACAAGTATCAACAGCAGCTGGGAAGCAAGAAGATAGAGCCTGAGCAC
EPAS1 CDS Otocolobus manul  ....................................................................................................
EPAS1 CDS Felis chaus       ....................T...............................................................................
EPAS1 CDS Felis nigripes    .....................A..............................................................................

                                     1810      1820      1830      1840      1850      1860      1870      1880      1890      1900
                            ....|....|....|....|....|....|....|....|....|....|....|....|....|....|....|....|....|....|....|....|
EPAS1 CDS Felis catus       CGGCCCCTGTCCTCCATCTTCTTTGATGGCGGCAGTAAAGTGTCCCTGCCCCCGTGCTGTGGCCAAGCCGGCACCCCTCTCTCCTCCCTGGGAGGCAGGT
EPAS1 CDS Otocolobus manul  .....G........................A.....................................................................
EPAS1 CDS Felis chaus       ....................................................................................................
EPAS1 CDS Felis nigripes    ....................................................................................................

                                     1910      1920      1930      1940      1950      1960      1970      1980      1990      2000
                            ....|....|....|....|....|....|....|....|....|....|....|....|....|....|....|....|....|....|....|....|
EPAS1 CDS Felis catus       CCAGCAGCACACAGTGGCCCCCTGATCCGCCATTACATTTTGGGCCCACGAAGTGGCCTGTTGTGGATCAGCACACGGAGTCCTTGGGGCCATCACCGTT
EPAS1 CDS Otocolobus manul  ..........................................................................G.........................
EPAS1 CDS Felis chaus       ..........................................................................G.........................
EPAS1 CDS Felis nigripes    ............................A...............................C.............G.........................

                                     2010      2020      2030      2040      2050      2060      2070      2080      2090      2100
                            ....|....|....|....|....|....|....|....|....|....|....|....|....|....|....|....|....|....|....|....|
EPAS1 CDS Felis catus       GGGGGCCCCCGTCAACTCGCCCCATCTCTCCGTCTTCAAGAAAAAGCTTGGCAGGTCTGCAAAAGGCTTTGGGCCCCAGGGCCCAGATGTGATGAGCCCG
EPAS1 CDS Otocolobus manul  ....................................................................................G...............
EPAS1 CDS Felis chaus       ..................A.................................................................G...............
EPAS1 CDS Felis nigripes    ....................................................................................G...............

                                     2110      2120      2130      2140      2150      2160      2170      2180      2190      2200
                            ....|....|....|....|....|....|....|....|....|....|....|....|....|....|....|....|....|....|....|....|
EPAS1 CDS Felis catus       GCCATGGTAGCCCTGTCCAACAAGCTGAAGCTGAAGCGACAGCGAGAATATGAGGAGCAGGCCTTCCAGGACCTGAGCGGGATCCAGGGGGACCCATCAG
EPAS1 CDS Otocolobus manul  ....................................................................................................
EPAS1 CDS Felis chaus       ........................................................A...........................................
EPAS1 CDS Felis nigripes    ......T.............................................................................................

                                     2210      2220      2230      2240      2250      2260      2270      2280      2290      2300
                            ....|....|....|....|....|....|....|....|....|....|....|....|....|....|....|....|....|....|....|....|
EPAS1 CDS Felis catus       GCAGCAGCACTTCACATCAGATGTGGAAAAGGATGAAGAGCCTCAGGGGCAGCGTGAACTGCCCTTTGGTGCCCGACAAGTTGCTGAGCGCAAGCGCCCC
EPAS1 CDS Otocolobus manul  ....................................................................................................
EPAS1 CDS Felis chaus       ....................................................................................................
EPAS1 CDS Felis nigripes    ....................................................................................................

                                     2310      2320      2330      2340      2350      2360      2370      2380      2390      2400
                            ....|....|....|....|....|....|....|....|....|....|....|....|....|....|....|....|....|....|....|....|
EPAS1 CDS Felis catus       CAGTGATGAGTTCACCCAACTTCCCATGAGGGGTGCGGGACAGCCTCTGAGACATCTGCCGCCGTCTGCCATGAGCCCCAGGGAGAACACCAAGAGTGGG
EPAS1 CDS Otocolobus manul  ....................................................................................................
EPAS1 CDS Felis chaus       ...............T....................................................................................
EPAS1 CDS Felis nigripes    ...........................A................................A.......................................

                                     2410      2420      2430      2440      2450      2460      2470      2480      2490      2500
                            ....|....|....|....|....|....|....|....|....|....|....|....|....|....|....|....|....|....|....|....|
EPAS1 CDS Felis catus       TTCCCCCCACAGTGCTACGCCCCCCAGTACCAGGACTACAGCCTGCCGTCAGCTCCCAAGGTGTCAGGCATGGCGAGTCGGCTGCTTGGGCCCTCCTTTG
EPAS1 CDS Otocolobus manul  .....................T....................................................A.........................
EPAS1 CDS Felis chaus       ......................................................................................C.............
EPAS1 CDS Felis nigripes    ....................................................................................................

                                     2510      2520      2530      2540      2550      2560      2570      2580      2590      2600
                            ....|....|....|....|....|....|....|....|....|....|....|....|....|....|....|....|....|....|....|....|
EPAS1 CDS Felis catus       AGCCCTACCTGCTGCCCGAATTGACCAGATATGACTGTGAGGTGAATGTTCCTGTGCCAGGAAGCTCCACGCTCCTGCAAGGAGGGGACCTCCTCAGAGC
EPAS1 CDS Otocolobus manul  ....................................................................................................
EPAS1 CDS Felis chaus       ....................................................................................................
EPAS1 CDS Felis nigripes    ....................................................................................................

                                     2610
                            ....|....|....|....
EPAS1 CDS Felis catus       CCTGGACCAGGCCACCTGA
EPAS1 CDS Otocolobus manul  ...................
EPAS1 CDS Felis chaus       ...................
EPAS1 CDS Felis nigripes    ...................
```

B) EPAS1 protein sequence

```
                              10        20        30        40        50        60        70        80        90       100
                      ....|....|....|....|....|....|....|....|....|....|....|....|....|....|....|....|....|....|....|....|
EPAS1 Felis catus     MTTDKEKKRSSSERRKEKSRDAARCRRSKETEVFYELAHELPLPHSVSSHLDKASIMRLAISFLRTHKLLSSVCSENESEAEANQQMDNLYLKALEGFIA
EPAS1 Otocolobus manul --------...........................................................................H................
EPAS1 Felis chaus     --------............................................................................................
EPAS1 Felis nigripes  --------............................................................................................

                             110       120       130       140       150       160       170       180       190       200
                      ....|....|....|....|....|....|....|....|....|....|....|....|....|....|....|....|....|....|....|....|
EPAS1 Felis catus     VVTQDGDMIFLSENISKFMGLTQVELTGHSIFDFTHPCDHEEIRENLSLKNGSGFGKKGKDMSTERDFFMRMKCTVTNRGRTVNLKSATWKVLHCTGQVK
EPAS1 Otocolobus manul ....................................................................................................
EPAS1 Felis chaus     ....................................................................................................
EPAS1 Felis nigripes  ....................................................................................................

                             210       220       230       240       250       260       270       280       290       300
                      ....|....|....|....|....|....|....|....|....|....|....|....|....|....|....|....|....|....|....|....|
EPAS1 Felis catus     VYNSCPPHSSLCSFKEPLLSCLIIMCEPIQHPSHMDIPLDSKTFLSRHSMDMKFTYCDDRITELIGYHPEELLGRSAYEFYHALDSENMTKSHQNLCTKG
EPAS1 Otocolobus manul ....................................................................................................
EPAS1 Felis chaus     ....................................................................................................
EPAS1 Felis nigripes  ....................................................................................................

                             310       320       330       340       350       360       370       380       390       400
                      ....|....|....|....|....|....|....|....|....|....|....|....|....|....|....|....|....|....|....|....|
EPAS1 Felis catus     QVVSGQYRMLAKHGGYVWLETQGTVIYNPRNLQPQCIMCVNYVLSEIEKNDVVFSMDQTESLFKPHLTAMSGIFDSSSEVAVSEQSDYLFTKLKEEPEEL
EPAS1 Otocolobus manul .......................................................................S............................
EPAS1 Felis chaus     .......................................................................S............................
EPAS1 Felis nigripes  .......................................................................S............................

                             410       420       430       440       450       460       470       480       490       500
                      ....|....|....|....|....|....|....|....|....|....|....|....|....|....|....|....|....|....|....|....|
EPAS1 Felis catus     AQLAPTAGDAIIALDFGSQNFEKSSAYSSALLPPSQPWSRELRSHSTQSEAGSLPAFTVPQAAASGSATPSATSSSSCSTPSSPGDYYTSLNDDLKIEVI
EPAS1 Otocolobus manul .............................................G......................................................
EPAS1 Felis chaus     ....................................................................................................
EPAS1 Felis nigripes  ....................................................................................................

                             510       520       530       540       550       560       570       580       590       600
                      ....|....|....|....|....|....|....|....|....|....|....|....|....|....|....|....|....|....|....|....|
EPAS1 Felis catus     EKLFTIDTEAKDQGSTQTDFSELDLETLAPYIPMDGEDFQLSPICPEERLLQEKPQSTPQHCFSTMTNIFQPLAPGASHSPFLLDKYQQQLGSKKIEPEH
EPAS1 Otocolobus manul ....................................................................................................
EPAS1 Felis chaus     .........................................................................V..........................
EPAS1 Felis nigripes  ....................................................................................................

                             610       620       630       640       650       660       670       680       690       700
                      ....|....|....|....|....|....|....|....|....|....|....|....|....|....|....|....|....|....|....|....|
EPAS1 Felis catus     RPLSSIFFDGGSKVSLPPCCGQAGTPLSSLGGRSSSTQWPPDPPLHFGPTKWPVVDQHTESLGPSPLGAPVNSPHLSVFKKKLGRSAKGFGPQGPDVMSP
EPAS1 Otocolobus manul ..........S...............................................A.........................................
EPAS1 Felis chaus     ..........................................................A.........................................
EPAS1 Felis nigripes  .....................................................A....A.........................................

                             710       720       730       740       750       760       770       780       790       800
                      ....|....|....|....|....|....|....|....|....|....|....|....|....|....|....|....|....|....|....|....|
EPAS1 Felis catus     AMVALSNKLKLKRQREYEEQAFQDLSGIQGDPSGSSTSHQMWKRMKSLRGSVNCPLVPDKLLSASAPSDEFTQLPMRGAGQPLRHLPPSAMSPRENTKSG
EPAS1 Otocolobus manul ....................................................................................................
EPAS1 Felis chaus     ....................................................................................................
EPAS1 Felis nigripes  ..L...........................................................................I........................

                             810       820       830       840       850       860       870
                      ....|....|....|....|....|....|....|....|....|....|....|....|....|....|...
EPAS1 Felis catus     FPPQCYAPQYQDYSLPSAPKVSGMASRLLGPSFEPYLLPELTRYDCEVNVPVPGSSTLLQGGDLLRALDQAT*
EPAS1 Otocolobus manul .......S................................................................*
EPAS1 Felis chaus     ........................................................................*
EPAS1 Felis nigripes  ........................................................................*
```

C) *EPAS1* 5´UTR

```
                                      10        20        30        40        50        60        70        80        90       100
                             ....|....|....|....|....|....|....|....|....|....|....|....|....|....|....|....|....|....|....|....|
EPAS1 5UTR Felis catus       GCGGCCGCGGGAGCCGGACATTCGAGAGCGGTCCGCGACACAGTCCCTGCACCCAACACAGCTCTAGCCTGAGGAGGCTGTACAATCCTCGGCAGTGTCC
EPAS1 5UTR Otocolobus manul  ....................................................................................................
EPAS1 5UTR O manul alt       ....................................................................................................
EPAS1 5UTR Felis chaus       ....................................................................................................
EPAS1 5UTR Felis nigripes    ....................................................................................................

                                     110       120       130       140       150       160       170       180       190       200
                             ....|....|....|....|....|....|....|....|....|....|....|....|....|....|....|....|....|....|....|....|
EPAS1 5UTR Felis catus       TGAGACTGTACGGTCATCTCCGCGTACGCGCCTGCCGGGCCCTGGACTCTTCCAACTCCCATCGTTGCAGCCACTTTTTCTTCTCTCTCTGTTTTCTTTT
EPAS1 5UTR Otocolobus manul  ....................................................................................................
EPAS1 5UTR O manul alt       ....................................................................................................
EPAS1 5UTR Felis chaus       ....................................................................................................
EPAS1 5UTR Felis nigripes    ....................................................................................................

                                     210       220       230       240       250       260       270       280       290       300
                             ....|....|....|....|....|....|....|....|....|....|....|....|....|....|....|....|....|....|....|....|
EPAS1 5UTR Felis catus       CTTTTCCTTCCTT---TTTTTTTTTTTTTTTTTTTTTTGAAAACCCAGAAAAGTGACTCTTCTTCGTGGGAAAAAGGAACTTCGGTCCCGCTCTCTCTGC
EPAS1 5UTR Otocolobus manul  .........T...TTT....C..............AA...............................................................
EPAS1 5UTR O manul alt       .........T...TTT....C-.............AA...............................................................
EPAS1 5UTR Felis chaus       .............CCT....................................................................................
EPAS1 5UTR Felis nigripes    .............----------.............................................................................

                                     310       320       330       340       350       360       370       380       390       400
                             ....|....|....|....|....|....|....|....|....|....|....|....|....|....|....|....|....|....|....|....|
EPAS1 5UTR Felis catus       CCTCTTTTTGGATTTGACAGCCTCCTCCCACTCCTTTTCCCGACCCCGCCTCCCTGTGCAGGTTCCTCCCAGTCACCTTTCTCCACCCTCCTCCCCCGCA
EPAS1 5UTR Otocolobus manul  .........................A..........................................................................
EPAS1 5UTR O manul alt       .........................A..........................................................................
EPAS1 5UTR Felis chaus       ....................................................................................................
EPAS1 5UTR Felis nigripes    ....................................................................................................

                                     410       420       430       440       450       460       470       480       490       500
                             ....|....|....|....|....|....|....|....|....|....|....|....|....|....|....|....|....|....|....|....|
EPAS1 5UTR Felis catus       CCTACCCAGCCTCCCGCAAACTTCCACCTGACGGTGCGGGGCGCCCGGGACCTGTGAGCACCTGGGACCTTTAACACCGGCCCCGGCCGCGAGGAGTTGG
EPAS1 5UTR Otocolobus manul  ................................C...................................................................
EPAS1 5UTR O manul alt       ................................C...................................................................
EPAS1 5UTR Felis chaus       ................................T...................................................................
EPAS1 5UTR Felis nigripes    ................................C...................................................................

                                     510       520       530       540       550       560       570       580
                             ....|....|....|....|....|....|....|....|....|....|....|....|....|....|....|....|....
EPAS1 5UTR Felis catus       CGAGGGCCACAGCGAAGGCGGCGCCTGCAATCCCCACCGGCCACGGAGCCCAGGTGTCCGGCGTCTCAGAGCACCACGGCGACA
EPAS1 5UTR Otocolobus manul  ....................................................................................
EPAS1 5UTR O manul alt       ....................................................................................
EPAS1 5UTR Felis chaus       ....................................................................................
EPAS1 5UTR Felis nigripes    ....................................................................................
```

D) *EPAS1* 3´UTR

```
                                       10        20        30        40        50        60        70        80        90       100
                               ....|....|....|....|....|....|....|....|....|....|....|....|....|....|....|....|....|....|....|....|
EPAS1 3UTR Felis catus         GCCAGGGCCCTCCACTGGGCAGGCCCCCCGCCCCCCC-TGCCCGCGCCGCCCCACCAGCTTCACTCTCTACGTCTATTTTTGCAACTAGGTATTTCTAAC
EPAS1 3UTR Otocolobus manul    .....................................-..............................................................
EPAS1 3UTR O manul alt         .....................................-..............................................................
EPAS1 3UTR Felis chaus         .....................................-..............................................................
EPAS1 3UTR Felis nigripes      .....................................C................T.............................................

                                      110       120       130       140       150       160       170       180       190       200
                               ....|....|....|....|....|....|....|....|....|....|....|....|....|....|....|....|....|....|....|....|
EPAS1 3UTR Felis catus         ACCAACACACTTTTTACAAGATGTACCTACCTGGTGAACTCGCCCACGTCCCCACGTGGTGGCCTTTTTCTAAAGATGCTCAC--TTTAGTGTATTTTTA
EPAS1 3UTR Otocolobus manul    ...................................................................................--...............
EPAS1 3UTR O manul alt         ...................................................................................--...............
EPAS1 3UTR Felis chaus         ...................................................................................AC...............
EPAS1 3UTR Felis nigripes      ...................................................................................--...............

                                      210       220       230       240       250       260       270       280       290       300
                               ....|....|....|....|....|....|....|....|....|....|....|....|....|....|....|....|....|....|....|....|
EPAS1 3UTR Felis catus         AGATGCACAGTTGTTTTACCTGCTGTGTTTTATTCTGTCAATGAAACGTTCTTAAATTTTGTAAGATTTTTCTTCCCCCGACTTTGATTACTTCTAATTT
EPAS1 3UTR Otocolobus manul    ....................................................................................................
EPAS1 3UTR O manul alt         ....................................................................................................
EPAS1 3UTR Felis chaus         ....................................................................................................
EPAS1 3UTR Felis nigripes      ..............................................................................T.....................

                                      310       320       330       340       350       360       370       380       390       400
                               ....|....|....|....|....|....|....|....|....|....|....|....|....|....|....|....|....|....|....|....|
EPAS1 3UTR Felis catus         ATATTATTCATGAGTCCCTCTGTCTCGCTCTCTCACACACACGATATCCATACTAACGAGTTTTGTGTACATTTGTTCTCTTGTAGGGAAAGCTTTGGCT
EPAS1 3UTR Otocolobus manul    ............G......................................C.....A..........................................
EPAS1 3UTR O manul alt         ............G......................................C.....A..........................................
EPAS1 3UTR Felis chaus         ....................................................................................................
EPAS1 3UTR Felis nigripes      ....................................................................................................

                                      410       420       430       440       450       460       470       480       490       500
                               ....|....|....|....|....|....|....|....|....|....|....|....|....|....|....|....|....|....|....|....|
EPAS1 3UTR Felis catus         TCATTTAACTAAAAAGGTCTTG---TTGTTGTTGTTGTTGTTGCCAAAGAGAAACAAAATAATTTTGCTTTCCAAGCTTGATTTTCATGGCCTCTTCCCT
EPAS1 3UTR Otocolobus manul    ......................---------.....................................................................
EPAS1 3UTR O manul alt         ......................---------.....................................................................
EPAS1 3UTR Felis chaus         ......................GTG...........................................................................
EPAS1 3UTR Felis nigripes      ......................G------.......................................................................

                                      510       520       530       540       550       560       570       580       590       600
                               ....|....|....|....|....|....|....|....|....|....|....|....|....|....|....|....|....|....|....|....|
EPAS1 3UTR Felis catus         CTCAAAGCCCTTCCTTCTTTTTTAAAACTAATCACCATATAATAAATTTCCATCTTTTCTTCTTTTCTTAAGCTGACTCTCGGCTCTAATTTTGACACAA
EPAS1 3UTR Otocolobus manul    ......................-.............................................................................
EPAS1 3UTR O manul alt         ......................-.............................................................................
EPAS1 3UTR Felis chaus         ....................................................................................................
EPAS1 3UTR Felis nigripes      ....................................................................................................

                                      610       620       630       640       650       660       670       680       690       700
                               ....|....|....|....|....|....|....|....|....|....|....|....|....|....|....|....|....|....|....|....|
EPAS1 3UTR Felis catus         CTGTTGGGGAAAAGGAAAATGCAAAGGGTGGGCTCCAGCATATGGGGTTAACTGTGAAGGTTGTGTAGTGTGGCTTTTCCCCCAGTGTGGTTTTTCTCCC
EPAS1 3UTR Otocolobus manul    ...........................................................................................G...T....
EPAS1 3UTR O manul alt         ...........................................................................................G...T....
EPAS1 3UTR Felis chaus         ....C...............................................................................................
EPAS1 3UTR Felis nigripes      ..A...............C.................................................................................

                                      710       720       730       740       750       760       770       780       790       800
                               ....|....|....|....|....|....|....|....|....|....|....|....|....|....|....|....|....|....|....|....|
EPAS1 3UTR Felis catus         GCATTCAGTGGATTTGTTTGGTAATTATTATTCAAAAATATAAGAGTTCTTTAAAAAGAAAAGTTATATCTGGGTTAAGTGTTTATCATATATATGGGTA
EPAS1 3UTR Otocolobus manul    ....................................................................................................
EPAS1 3UTR O manul alt         ....................................................................................................
EPAS1 3UTR Felis chaus         ....................................................................................................
EPAS1 3UTR Felis nigripes      ......................G.............................................................................

                                      810       820       830       840       850       860       870       880       890       900
                               ....|....|....|....|....|....|....|....|....|....|....|....|....|....|....|....|....|....|....|....|
EPAS1 3UTR Felis catus         CTTTGTAATATCTAAAAACTTGGAAATGGAATCCTGCTCACGAAATCACTTTAAGATCTTTTTGAAGCTGTCCATTTTCTCTCCCCTGGTGTTGCTGACA
EPAS1 3UTR Otocolobus manul    ....................................................................................................
EPAS1 3UTR O manul alt         ....................................................................................................
EPAS1 3UTR Felis chaus         ....................................................................................................
EPAS1 3UTR Felis nigripes      ....................................................................................................

                                      910       920       930       940       950       960       970       980       990      1000
                               ....|....|....|....|....|....|....|....|....|....|....|....|....|....|....|....|....|....|....|....|
EPAS1 3UTR Felis catus         CTGCAGATTGTACAGAACTCCCACGGGCCTGTACCAGCACTGCTCAAGGCCTCTTGCTGGTGGCTTTTTGCTTCGGGGATATGCCCTGGGCATGACAGAC
EPAS1 3UTR Otocolobus manul    ....................................................................................................
EPAS1 3UTR O manul alt         ....................................................................................................
EPAS1 3UTR Felis chaus         ....................................................................................................
EPAS1 3UTR Felis nigripes      .......................T............................................................................

                                     1010      1020      1030      1040      1050      1060      1070      1080      1090      1100
                               ....|....|....|....|....|....|....|....|....|....|....|....|....|....|....|....|....|....|....|....|
EPAS1 3UTR Felis catus         AATCCAAGACGTGGAATCATAAAGTGTGGGAGCACTGCGAGCTGTCTTCTCGTGTTCTATATGTATTATGTATGTATGTATGTATGTATATTATTATTGC
EPAS1 3UTR Otocolobus manul    ....................................................................................................
EPAS1 3UTR O manul alt         ....................................................................................................
EPAS1 3UTR Felis chaus         ....................................................................................................
EPAS1 3UTR Felis nigripes      ....................................................................................................

                                     1110      1120      1130      1140      1150      1160      1170      1180      1190      1200
                               ....|....|....|....|....|....|....|....|....|....|....|....|....|....|....|....|....|....|....|....|
EPAS1 3UTR Felis catus         TGCCAAGAGGGTCTGATGGCATGTCATGGGGTGGGGGTGAGGGTGGGGCAAATCTAAGGGAGGGGAAGTGCTTTAATTTTTCTTCAGATTTTGTTGCCAG
EPAS1 3UTR Otocolobus manul    ..................................................................................................G.
EPAS1 3UTR O manul alt         ..................................................................................................G.
EPAS1 3UTR Felis chaus         ....................................................................................................
EPAS1 3UTR Felis nigripes      ....................................................................................................
```

```
                                    1210      1220      1230      1240      1250      1260      1270      1280      1290      1300
                                ....|....|....|....|....|....|....|....|....|....|....|....|....|....|....|....|....|....|....|....|
EPAS1 3UTR Felis catus          CCCTTCAAGTGCACTGAGCTATGGGACCCAAAAGGTCTTTCACATGGCACATTTGGCTATTTCCAGAATTACCACGAGACGGTTTTGGTGGGAGTTCATG
EPAS1 3UTR Otocolobus manul     ........................................................A......................T....................
EPAS1 3UTR O manul alt          ........................................................A......................T....................
EPAS1 3UTR Felis chaus          ........................................................A.................T.........................
EPAS1 3UTR Felis nigripes       .........................................................................C..........................

                                    1310      1320      1330      1340      1350      1360      1370      1380      1390      1400
                                ....|....|....|....|....|....|....|....|....|....|....|....|....|....|....|....|....|....|....|....|
EPAS1 3UTR Felis catus          AAAGACGGGATTCAGAAATGGGGGTCGTGATCCAGCCATGGTGCTCCCAGCTTACCAGCCTGGAGCACATGTTGCTGGCCTGAGAGAGAATATGGGAAGG
EPAS1 3UTR Otocolobus manul     ......A.....................................................G.......................................
EPAS1 3UTR O manul alt          ......A.....................................................G.......................................
EPAS1 3UTR Felis chaus          ......A....................................................TG.......................................
EPAS1 3UTR Felis nigripes       ......A.....................................................A.......................................

                                    1410      1420      1430      1440      1450      1460      1470      1480      1490      1500
                                ....|....|....|....|....|....|....|....|....|....|....|....|....|....|....|....|....|....|....|....|
EPAS1 3UTR Felis catus          GGG--CACAGCTGGTCCTGGCCGGCCACGCACCCCTTCCTCAGCCTGCCGTTAGGTGGCCCAGCAAGGAATAGCAGGCAGACTTCCTGGGTGGCCCCTAG
EPAS1 3UTR Otocolobus manul     ...G-..............A................................................................................
EPAS1 3UTR O manul alt          ...GG..............A................................................................................
EPAS1 3UTR Felis chaus          ...--..............A..A.............................................................................
EPAS1 3UTR Felis nigripes       ...--..............A................................C...............................T.....G.........

                                    1510      1520      1530      1540      1550      1560      1570      1580      1590      1600
                                ....|....|....|....|....|....|....|....|....|....|....|....|....|....|....|....|....|....|....|....|
EPAS1 3UTR Felis catus          CCTCAGGCCTTCCCCAAGTTCTGTCTGTCACCTGAAGTGACTTATTTAGGTAGGAAGCACTGAAAATCAAGTGTTCTCAGAGCACTTTGTAACTCACTGG
EPAS1 3UTR Otocolobus manul     ...............................................................................................C....
EPAS1 3UTR O manul alt          ...............................................................................................C....
EPAS1 3UTR Felis chaus          ......................C.............................................................................
EPAS1 3UTR Felis nigripes       ....................................................................................................

                                    1610      1620      1630      1640      1650      1660      1670      1680      1690      1700
                                ....|....|....|....|....|....|....|....|....|....|....|....|....|....|....|....|....|....|....|....|
EPAS1 3UTR Felis catus          GTAAGAAGGACGATACCTTTTTGGTTTTTAAATACCAATCACATGGAGCTTTTCTGTCCTGGAGACAACAAGGAAGTTTCTAGAAACACACA----GCAC
EPAS1 3UTR Otocolobus manul     ...........A...................................................T............................CAAA....
EPAS1 3UTR O manul alt          ...........A...................................................T............................CAAA....
EPAS1 3UTR Felis chaus          ...............................................................T............................CAAA....
EPAS1 3UTR Felis nigripes       ..........T....................................................T............................CAAA....

                                    1710      1720      1730      1740      1750      1760      1770      1780      1790      1800
                                ....|....|....|....|....|....|....|....|....|....|....|....|....|....|....|....|....|....|....|....|
EPAS1 3UTR Felis catus          AGCAGGTGAAGAATTTGGTAAAGCTGGAGGGACATATTGCCAAAAACACAACAAAAAAAA-CAATTTCAAAAGAAATCTCTAAGAAGAATGACCCGGTCG
EPAS1 3UTR Otocolobus manul     ............................................................A.......................................
EPAS1 3UTR O manul alt          ............................................................A.......................................
EPAS1 3UTR Felis chaus          ............................................................A.......................................
EPAS1 3UTR Felis nigripes       ....................................C.......................-.......G...............................

                                    1810      1820      1830      1840      1850      1860      1870      1880      1890      1900
                                ....|....|....|....|....|....|....|....|....|....|....|....|....|....|....|....|....|....|....|....|
EPAS1 3UTR Felis catus          CTGCATTGACCAAATCACACTTTAGCACCACCGCTTTGCCCCACGCACGGCGGCAGAACTTGAAGGGTTACTGACGCGTAAACGCTGGTGTTTGGTGTTT
EPAS1 3UTR Otocolobus manul     ......................---...........................................................................
EPAS1 3UTR O manul alt          ......................---...........................................................................
EPAS1 3UTR Felis chaus          ................................................A...................................................
EPAS1 3UTR Felis nigripes       ................................A...................................................................

                                    1910      1920      1930      1940      1950      1960      1970      1980      1990      2000
                                ....|....|....|....|....|....|....|....|....|....|....|....|....|....|....|....|....|....|....|....|
EPAS1 3UTR Felis catus          GATTTCCTGTGTGTGTTGCCTCAGCATTAAGGGCATTTTTACCCTTGCAGTTTTACTAAAACACTCTGAAAAATATTCCAAGCTTCATAGTAACCTTACC
EPAS1 3UTR Otocolobus manul     ....................................................................................................
EPAS1 3UTR O manul alt          ....................................................................................................
EPAS1 3UTR Felis chaus          ....................................................................................................
EPAS1 3UTR Felis nigripes       ....................................................................................................

                                    2010      2020      2030      2040      2050      2060      2070      2080      2090      2100
                                ....|....|....|....|....|....|....|....|....|....|....|....|....|....|....|....|....|....|....|....|
EPAS1 3UTR Felis catus          TGTCAACGTAACGAGTTCATGAACATTATTGTATTGTCAAACTCCTACTGACAACATTATTACTATAGGGGAGCTTAACTTTATAAAGAAATGTATTTTG
EPAS1 3UTR Otocolobus manul     ....................................................................................................
EPAS1 3UTR O manul alt          ....................................................................................................
EPAS1 3UTR Felis chaus          ...............................................................C....................................
EPAS1 3UTR Felis nigripes       ...............................................................C....................................

                                    2110      2120      2130
                                ....|....|....|....|....|....|...
EPAS1 3UTR Felis catus          ACACTGATATCTTATTAAAGTGTTCTGATCCTA
EPAS1 3UTR Otocolobus manul     .................................
EPAS1 3UTR O manul alt          .................................
EPAS1 3UTR Felis chaus          .................................
EPAS1 3UTR Felis nigripes       .........C.......................
```

E) *EPAS1* intron 7 lncRNA

```
                                 10        20        30        40        50        60        70        80        90       100
                         ....|....|....|....|....|....|....|....|....|....|....|....|....|....|....|....|....|....|....|....|
lncRNA Felis catus       ACTGGGGGAGGAGAAACAGAGCCTGAGGTCAGGGAGGTTGGAAAGCGAGACCTCACAGGGAAGGCAGTCTGCGGGGAAACAGACAAGACTGGCCCCTAAG
lncRNA Otocolobus manul  .................G..................................................................................
lncRNA Felis chaus       ....................................................................................................
lncRNA Felis nigripes    ....................................................................................................

                                110       120       130       140       150       160       170       180       190       200
                         ....|....|....|....|....|....|....|....|....|....|....|....|....|....|....|....|....|....|....|....|
lncRNA Felis catus       GCAGCAAGGACCTGCTTATGGCTTCTTTCCTCCTCCAGATTGCTCTAAATGCTCATGTAGCATTTGTTTACTGTATGAAAGCCCTGGGAAGGTGTCTCAC
lncRNA Otocolobus manul  ...............................T..........T.................................................C.C.....
lncRNA Felis chaus       ................................T.........T.........................................................
lncRNA Felis nigripes    ..........................................T.........................................................

                                210       220       230       240       250       260       270       280       290       300
                         ....|....|....|....|....|....|....|....|....|....|....|....|....|....|....|....|....|....|....|....|
lncRNA Felis catus       ATTCCCTACTCCGGAATTCTTCATCCGCTCCCCGTAAAAAGGAATCAATAAATCTCCTTAAATTAAAGCCTAATGTTAGAATTTCTGGTAGTTTTCCAAC
lncRNA Otocolobus manul  .........................T......A...................................T...............................
lncRNA Felis chaus       .........................T......A...................................T...............................
lncRNA Felis nigripes    .........................T......A...................................T...............................

                                310       320       330       340       350       360       370       380       390       400
                         ....|....|....|....|....|....|....|....|....|....|....|....|....|....|....|....|....|....|....|....|
lncRNA Felis catus       CTGGACATATACATTTTCATTCCTTGGGAATTCATTCTTCAGAATCCTAGCTCAGAACTCAAATCACAGAGAACTGGAAGAGGCACAGTGTAAGCATTAG
lncRNA Otocolobus manul  ....................................................................................................
lncRNA Felis chaus       ....................................................................................................
lncRNA Felis nigripes    ..........................................................................................C.........

                                410       420       430       440       450       460       470       480       490       500
                         ....|....|....|....|....|....|....|....|....|....|....|....|....|....|....|....|....|....|....|....|
lncRNA Felis catus       AAAATCTTGCTTCATGGGGACTTTGGGAAAAGAACAATCCCCCTTAGATTTGGTTTCCTTATCTGCAAAATGAGGTACTGATAAAGATAATAGTAATGAT
lncRNA Otocolobus manul  .....................................................................................A..............
lncRNA Felis chaus       .....................................................................................A..............
lncRNA Felis nigripes    .....................................................................................A..............

                                510       520       530       540       550       560       570       580       590       600
                         ....|....|....|....|....|....|....|....|....|....|....|....|....|....|....|....|....|....|....|....|
lncRNA Felis catus       AATAATCATTCTTAACTACCCGTATACCACAGCTATTCGTACTGGGCACGACATCTGCCTGTCTGGCTTATCAGGTGCAGAAGTGTTGACTGAATATCCT
lncRNA Otocolobus manul  ....................................................................................................
lncRNA Felis chaus       ....................................................................................................
lncRNA Felis nigripes    ..................................G.................................................................

                                610       620       630       640       650       660       670       680       690       700
                         ....|....|....|....|....|....|....|....|....|....|....|....|....|....|....|....|....|....|....|....|
lncRNA Felis catus       GGACAAACGCTGTATAGGAAAAGCCACCTCTTTCCCAGGTCTGCAGGTAACGTTGTGAGGGACCCCACGGAGAAGACCCAGATTCTCTGTAAACGCGGAG
lncRNA Otocolobus manul  .......T........................C..................A...........................................T....
lncRNA Felis chaus       .......T......C.................C...................................................................
lncRNA Felis nigripes    .......T........................C...................................................................

                                710       720       730       740       750       760       770       780       790       800
                         ....|....|....|....|....|....|....|....|....|....|....|....|....|....|....|....|....|....|....|....|
lncRNA Felis catus       AATTGCCCCCCGTTCTCTGGGTACGCTCACGGGCCACCTCCCCATTCACCAGCCCTGTGTGGTGGAGGAAGGCAGCGTGGTTCACGCTAACACCACTTGT
lncRNA Otocolobus manul  .............................T.........-....................................................T.......
lncRNA Felis chaus       .............................T......................................................................
lncRNA Felis nigripes    .............................T......................................................................

                                810       820       830       840       850       860       870       880       890       900
                         ....|....|....|....|....|....|....|....|....|....|....|....|....|....|....|....|....|....|....|....|
lncRNA Felis catus       GGTCTTTTACATTAATGTTTTTCTAGGGGCGCCTGGGTGGCGCAGTCGGTTAAGCGTCCGACTTCAGCCAGGTCACGATCTCGCAGTCCGTGAGTTCGAG
lncRNA Otocolobus manul  .........-------------------------------------------------------------------------------------------
lncRNA Felis chaus       .........-------------------------------------------------------------------------------------------
lncRNA Felis nigripes    .........-------------------------------------------------------------------------------------------

                                910       920       930       940       950       960       970       980       990      1000
                         ....|....|....|....|....|....|....|....|....|....|....|....|....|....|....|....|....|....|....|....|
lncRNA Felis catus       CCCCGCGTCGGGCTCTGGGCTGATGGCTCGGAGCCTGGAGCCTGTTTCCGATTCTGTGTCTCCCTCTCTCTCTGCCCCTCCCCCGTTCATGCTCTGTCTC
lncRNA Otocolobus manul  ----------------------------------------------------------------------------------------------------
lncRNA Felis chaus       ----------------------------------------------------------------------------------------------------
lncRNA Felis nigripes    ----------------------------------------------------------------------------------------------------

                               1010      1020      1030      1040      1050      1060      1070      1080      1090      1100
                         ....|....|....|....|....|....|....|....|....|....|....|....|....|....|....|....|....|....|....|....|
lncRNA Felis catus       TCTCGGTCCCAAAAATAAATAAACGTTGAAAAAAAAAATTATCATTAATGTTTTTCTAGGCACAGTTACCGTCTTCAAAAAAAAATTGAACAAGCACCAC
lncRNA Otocolobus manul  ------------------------------------------..................................----....................
lncRNA Felis chaus       ------------------------------------------..................................-.......................
lncRNA Felis nigripes    ------------------------------------------..........................................................

                               1110      1120      1130      1140      1150      1160      1170      1180      1190      1200
                         ....|....|....|....|....|....|....|....|....|....|....|....|....|....|....|....|....|....|....|....|
lncRNA Felis catus       CTCCAGGGTGCAGGAAATACTAACTTACGGGTGTGCAGCGCTTTGCAGTGGTCAAAGCCATCTCAAGATGAACGCGCTACTG-AAAGCAACTAGAAGTGC
lncRNA Otocolobus manul  ...........................................................................A..G...C.................
lncRNA Felis chaus       ........................................................................T..A..G...C.................
lncRNA Felis nigripes    ...........................................................................A..G...C.................

                               1210      1220      1230      1240      1250      1260      1270      1280      1290      1300
                         ....|....|....|....|....|....|....|....|....|....|....|....|....|....|....|....|....|....|....|....|
lncRNA Felis catus       CCGCACGTAAGTGATCAATGAGGAT--GTGTTATCAGCAGCTCATGTGACACATGGTGGCACCATGAAGTGTCTCCTGTGATTCCTGTTTTACATCTGAA
lncRNA Otocolobus manul  .............G...........GA.................................................C.......................
lncRNA Felis chaus       .........................--.........................G...............................................
lncRNA Felis nigripes    ......A..................--.........................................................................

                               1310      1320      1330      1340      1350      1360      1370      1380      1390      1400
                         ....|....|....|....|....|....|....|....|....|....|....|....|....|....|....|....|....|....|....|....|
lncRNA Felis catus       GAAATGGAAGGCTTCGAACTATTGAGTGACTTACTGAAAGGTCCCCTCGGTGGAAACAGTAGACGGGATCACAGCCTAAATTAAAGACTCCTAATCTAGG
lncRNA Otocolobus manul  ....C..-............................................................................................
lncRNA Felis chaus       ....C..-............................................................................................
lncRNA Felis nigripes    ....C..-............................................................................................
```

```
                                  .
lncRNA Felis catus                C
lncRNA Otocolobus manul           .
lncRNA Felis chaus                .
lncRNA Felis nigripes             .
```
