# Supplementary figures and images for: Four new genome sequences of the Pallas’s cat (Otocolobus manul): an insight into the patterns of within-species variability
Source: Front Genet. 2024 Dec 9;15:1463774. doi: 10.3389/fgene.2024.1463774 (PMC11667119; doi:10.3389/fgene.2024.1463774)

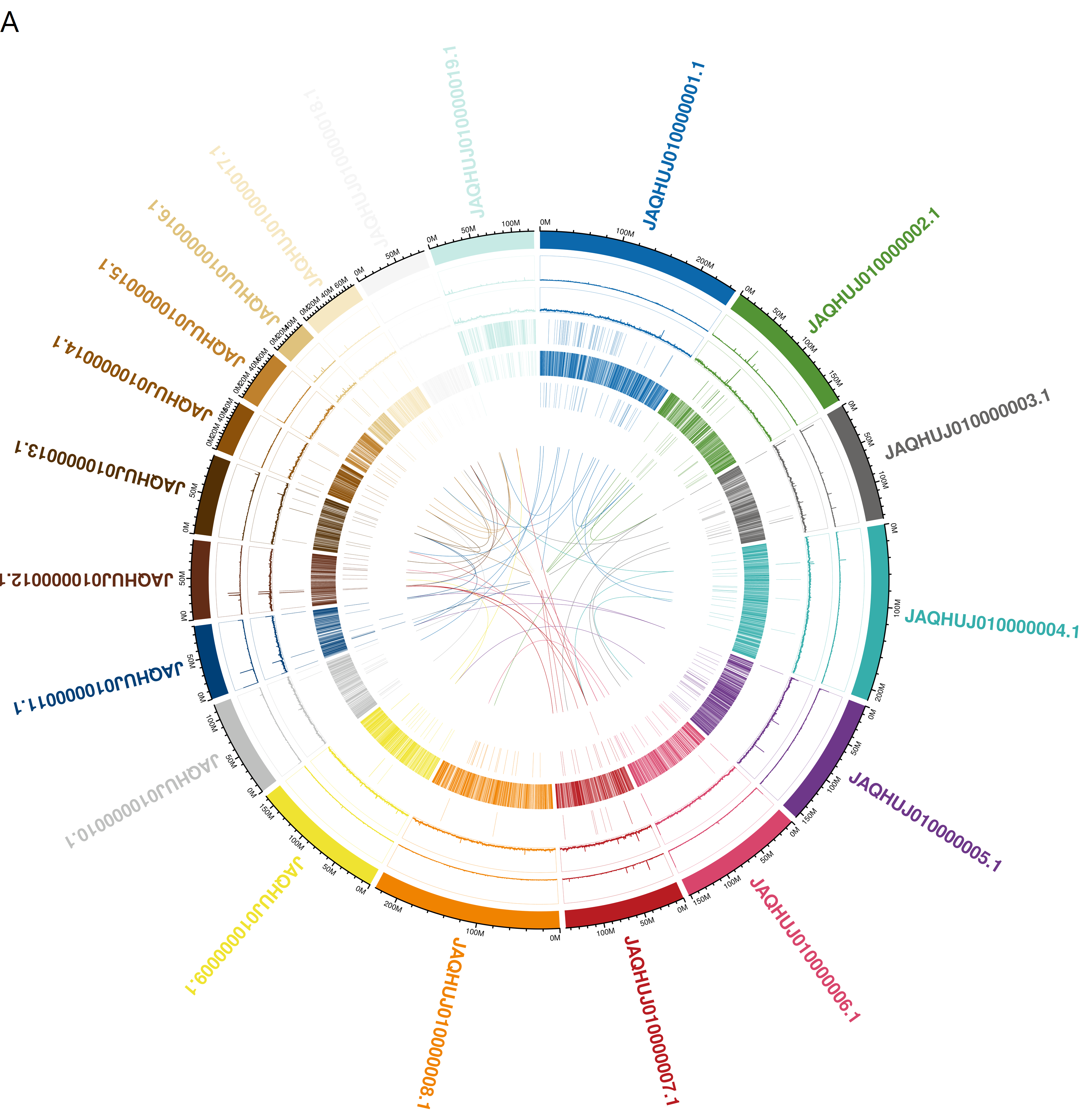

Supplement: Supplementary file 1 [file DataSheet1.zip › Supplementary Material/Supplementary Figure 1A.png]

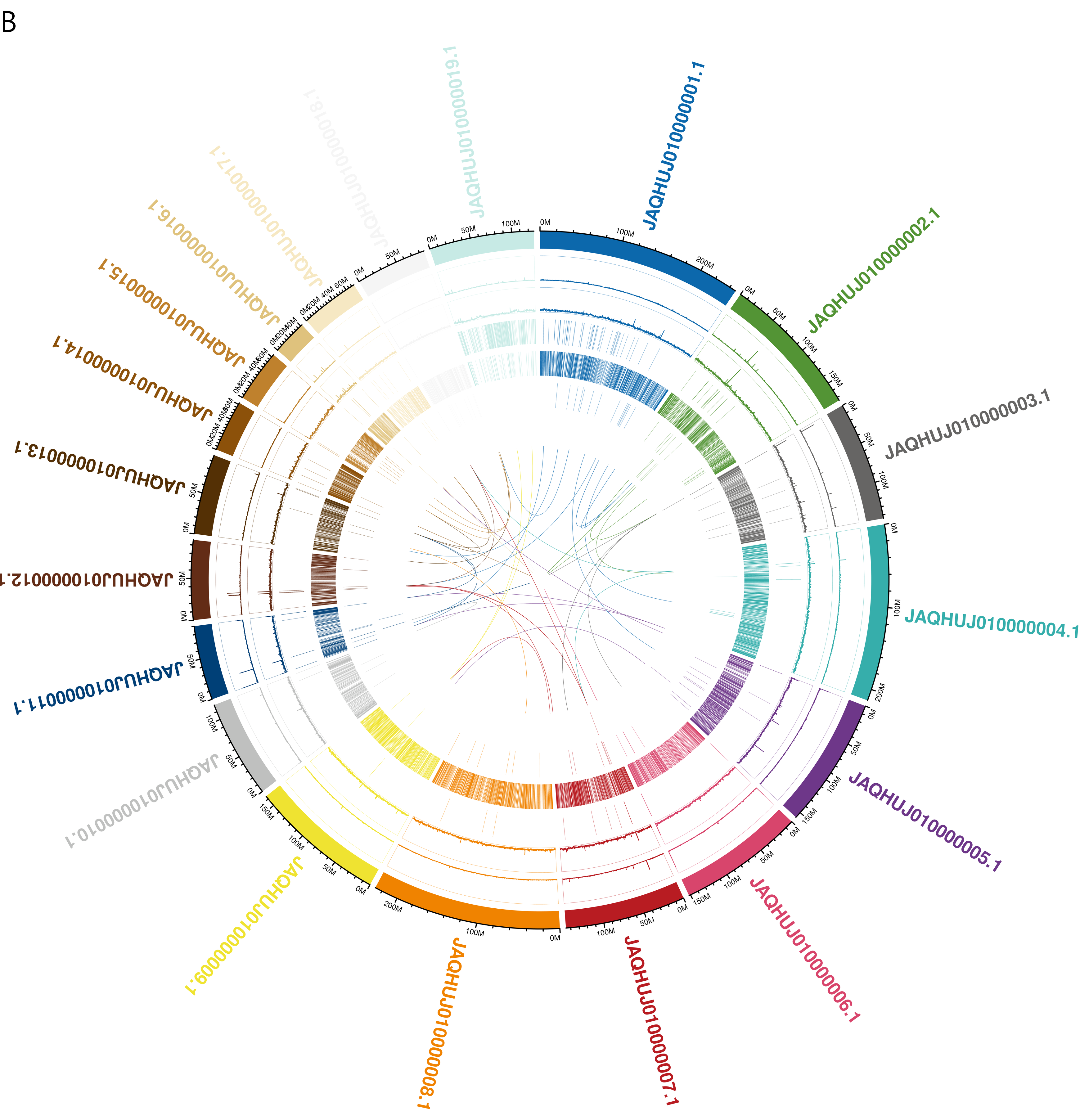

Supplement: Supplementary file 1 [file DataSheet1.zip › Supplementary Material/Supplementary Figure 1B.png]

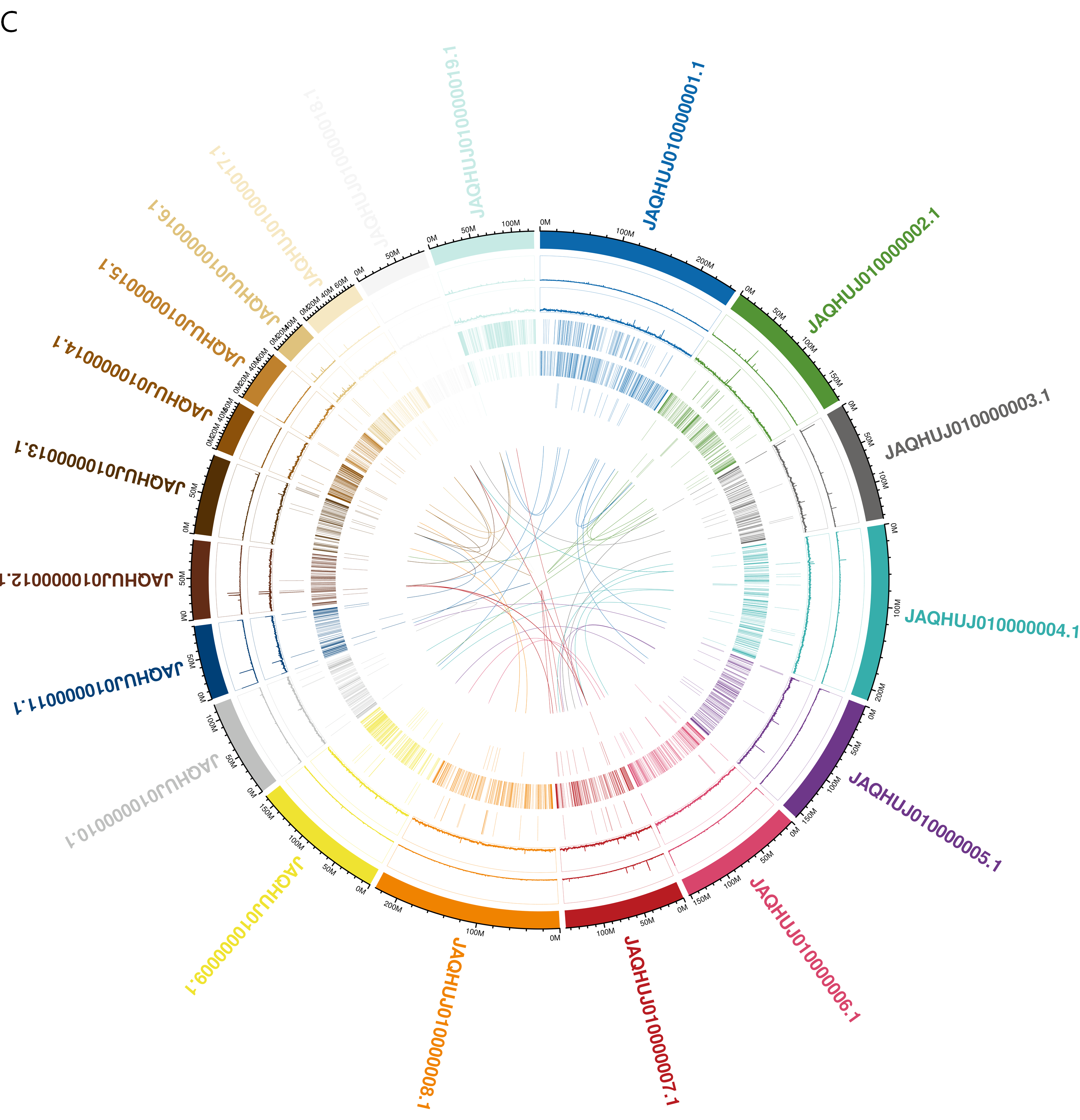

Supplement: Supplementary file 1 [file DataSheet1.zip › Supplementary Material/Supplementary Figure 1C.png]

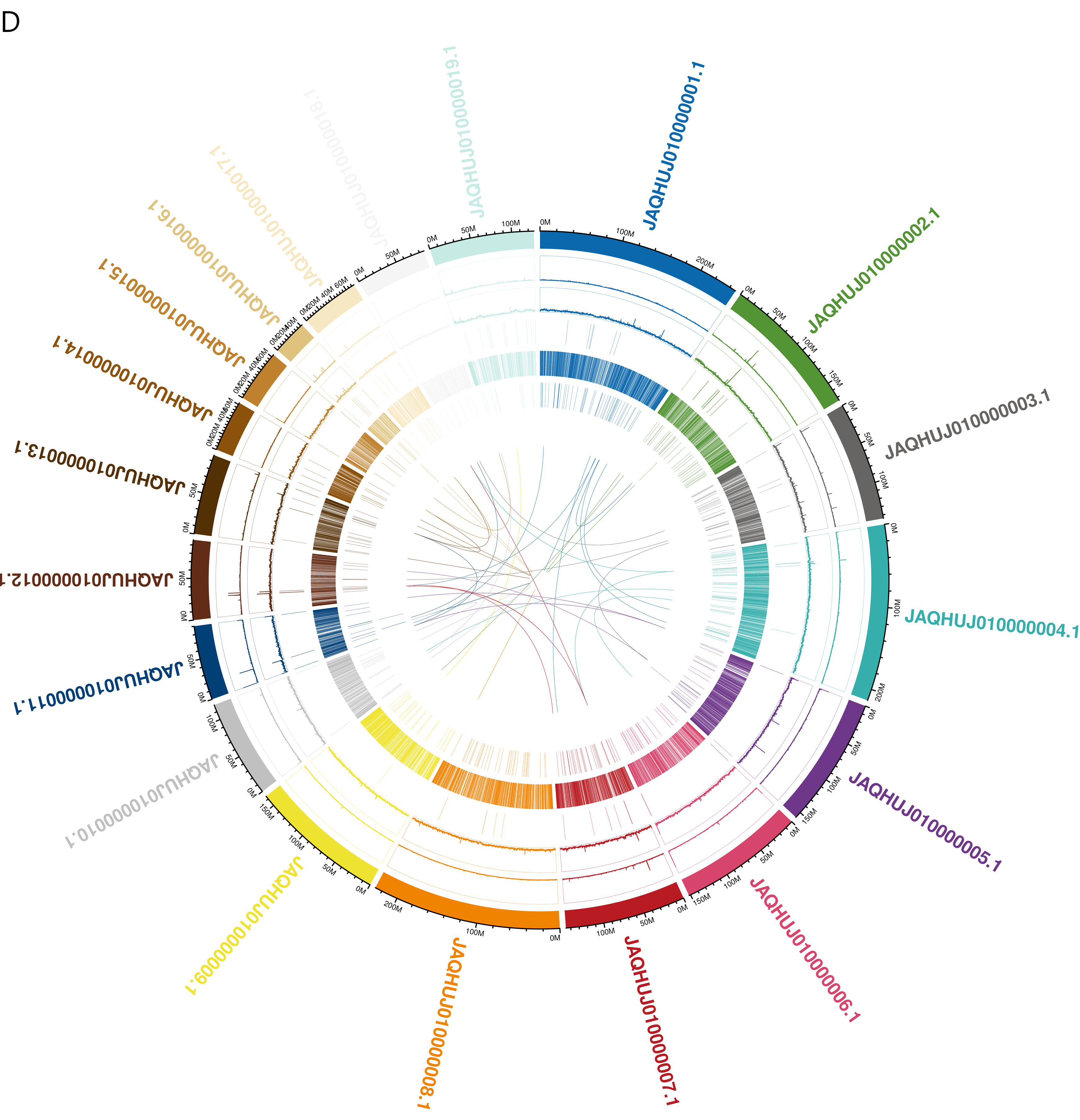

Supplement: Supplementary file 1 [file DataSheet1.zip › Supplementary Material/Supplementary Figure 1D.png]
